# ECM‐Stiffness Mediated Persistent Fibroblast Activation Requires Integrin and Formin Dependent Chromatin Remodeling

**DOI:** 10.1002/advs.202517631

**Published:** 2026-03-31

**Authors:** Swathi Packirisamy, Oscar André, Zhimeng Fan, Pontus Nordenfelt, Vinay S Swaminathan

**Affiliations:** ^1^ Division of Oncology Department of Clinical Sciences Lund University Lund Sweden; ^2^ Wallenberg Center for Molecular Medicine Lund University Lund Sweden; ^3^ Division of Infection Medicine Department of Clinical Sciences Lund University Lund Sweden

**Keywords:** integrin‐mediated mechanotransduction, nucleus‐actin coupling, persistent fibroblast activation, physical regulation of chromatin

## Abstract

Transient activation of fibroblasts into contractile myofibroblasts is essential for extracellular matrix (ECM) production and remodeling during wound healing and tissue regeneration. While ECM‐dependent mechanisms mediating transient activation is well studied, how fibroblasts switch from transient to a persistently activated state and drive fibrosis and aberrant tissue repair in diseases such as cancer is less understood. Here, we show that human cancer‐associated fibroblasts (CAFs) switch from transient to persistently activated states upon prolonged exposure to stiff ECMs and stiffness‐dependent secreted factors. This switch is accompanied by activation of ECM‐stiffness–dependent mechanotransduction pathways and changes in the nuclear architecture and its association with chromatin. Mechanistically, we identify two pathways required for this switch‐ ECM ligand binding and activation of β1 integrins smoothens the nuclear lamina during prolonged exposure and reduces lamin–chromatin contacts while in parallel, exposure to the stiff ECM activates the formin mammalian Diaphanous‐related formin 2 (mDia2) and independent of alterations in the nuclear architecture alters lamin–chromatin coupling, likely through its role in assembling nuclear actin. Importantly, we find that blocking either pathway prevents persistent myofibroblast activation, which is rescued by inhibition of histone deacetylases, indicating that dynamic chromatin modifications act downstream of these ECM‐dependent pathways to maintain the persistently activated state. These findings link integrin‐based ECM sensing to chromatin remodeling and fibroblast memory, with implications for stromal plasticity in the tumor microenvironment.

## Graphical abstract

Prolonged exposure to stiff extracellular matrix drives cancer‐associated fibroblasts into a persistently activated myofibroblast state. Two parallel pathways are identified: β1 integrin activation smoothens the nuclear lamina to reduce lamin–chromatin contacts, while the formin mDia2 regulates nuclear actin to alter chromatin organization. Both pathways converge on histone deacetylation, revealing an epigenetic basis for fibroblast mechanical memory.

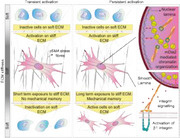

## 1 Introduction

The mechanical properties of the extracellular matrix (ECM) in the tumor microenvironment (TME), including its stiffness and architecture, are known drivers of cancer cell proliferation and metastasis [1, 2, 3]. Just as in normal tissues, these mechanical properties are primarily regulated by stromal cells including the resident fibroblasts which are called cancer associated fibroblasts (CAFs) in the TME [4, 5, 6]. In normal tissues, during processes such as wound healing, fibroblasts get transiently activated to myofibroblasts by cues like growth factors as well as changes in the mechanical properties of the ECM associated with tissue damage [7, 8, 9]. Once activated, myofibroblasts upregulate proliferation, secretion and contractility to produce and remodel the ECM required for tissue repair. Most importantly, upon completion of tissue repair and dissipation of activating cues, myofibroblasts either switch back to their quiescent state or undergo apoptosis, a process that is critical to maintain tissue homeostasis [10, 11]. Several recent single cell sequencing studies have revealed that there is a broad heterogeneity when it comes to CAF subtypes and functional states within the TME [5, 12, 13]. Independent of this heterogeneity, it has however been proposed that a subset of fibroblasts switch from transient to persistently activated states, i.e. lose their ability to deactivate, and continuously produce and remodel the ECM, significantly altering the mechanical properties of the TME and ultimately promote cancer cell growth and metastasis [14, 15]. Consistent with this, quiescent CAF populations have been identified in early‐stage tumors where they resemble normal fibroblasts [16, 17]. Thus, understanding mechanisms that induce the switch in plasticity of fibroblast activation from transient to persistently activated states is critical in understanding the mechanical regulation of tumor metastasis.

Several previous studies have highlighted the role of prolonged exposure to mechanical and biochemical cues in the switch between transient and persistent activation of fibroblasts and mesenchymal stem cells (MSCs) in the context of fibrosis [18, 19, 20]. It is currently understood that exposure to high ECM stiffness promotes transient activation of fibroblasts over extended periods which alters the epigenetic landscape by changing chromatin architecture and histone modifications and induces persistent activation. Specifically, ECM stiffness mediated alterations in histone acetylation and methylation as well as changes in chromatin packing have been shown to be vital for this switch [21, 22, 23, 24]. Unsurprisingly, several key physical components of the nucleus including the *LINC (Linker of Nucleoskeleton and Cytoskeleton) complex* that physically connects the cytoskeleton to the nuclear lamina and transmits mechanical forces from the ECM to the nucleus, *nuclear lamins* that form a meshwork underlying the inner nuclear membrane, as well as overall mechanical properties of the nucleus have emerged as important regulators of ECM stiffness mediated changes in chromatin organization and modifications [25, 26, 27]. However, the nucleus is indirectly connected to the ECM via integrin‐based focal adhesion (FA) complexes and the cytoskeleton network and less is known about the molecular identities and mechanisms of FA and cytoskeleton regulators mediating this loss of plasticity in fibroblast activation and corresponding epigenetic changes. In addition, the mechanisms by which physical changes in the nucleus, resulting from prolonged exposure to high ECM stiffness, are translated into specific chromatin modifications during this process remain poorly understood.

Here we report that CAFs along with stromal fibroblasts also exhibit a switch from transient to persistent myofibroblast state in response to prolonged exposure to a stiff ECM. This switch requires a threshold period of exposure and accumulation of secreted factors that is dependent on the magnitude and duration of exposure to the stiff ECM. We show that persistent activation is associated with chromatin remodeling at the nuclear lamina and is dependent on high level activation of β1 integrins and its regulation of nuclear YAP localization and smoothening of the nuclear lamina. Furthermore, we identify the actin nucleating formin mDia2, and its role in regulating actin assembly in the nucleus as essential in chromatin organization and persistent activation, independent of changes in nuclear morphology or perinuclear actin. Finally, we demonstrate that histone deacetylation acts downstream of both integrin and mDia2 pathways and is sufficient to drive persistent myofibroblast activation, even in the absence of large‐scale chromatin repositioning. Taken together, these findings uncover a pathway linking ECM mechanosensing to chromatin remodeling and persistent myofibroblast activation, with potential relevance to fibroblast plasticity in the TME and broader implication in fibrosis.

## 2 Results

### 2.1 Persistent Myofibroblast Activation Requires Prolonged Exposure to Stiff ECM and Stiffness‐Dependent Secreted Factors

To investigate the mechanisms underlying ECM stiffness mediated persistent myofibroblast activation, we first sought to determine the ECM stiffness that promotes maximum transient myofibroblast activation in human vulval cancer associated fibroblast (vCAF) cell line. To do this, we used fibronectin (FN) coated polyacrylamide (PA) hydrogels across a wide stiffness range (0.5, 1, 4, 8, 25 and 50 kPa) and plated vCAFs for 20 h (h) prior to fixing and staining with either α‐smooth muscle actin (αSMA) and Hoechst 33342 or with phalloidin, Hoechst 33342 and the transcription co‐factor YAP (Figure S1). Activated myofibroblasts were identified as cells that incorporated αSMA into stress fibres and quantified using a custom Python‐based image analysis pipeline. Per‐cell coherence values were computed from αSMA positive pixels and used to classify cells into activated (αSMA^+^), intermediate (αSMA^i^), or non‐activated (αSMA^−^) states where αSMA^+^ cells have highly aligned fibrous αSMA structures, αSMA^−^ cells show isotropic distributions of αSMA signal and αSMA^i^ cells have intermediate αSMA coherence. Cell spread area and nuclear to cytoplasmic ratio YAP (YAP N/C) were used as readouts for activation of ECM stiffness‐mediated mechanotransduction pathways [28, 29, 30].

As expected, on soft hydrogels (0.5 and 1 kPa), vCAFs showed limited cell spread area, low YAP N/C and diffused αSMA staining with few αSMA^+^ cells (Figure S1a). Increasing hydrogel stiffness resulted in an increase in cell spread area and YAP N/C while αSMA^+^ myofibroblasts remained low at 4 kPa with the highest fraction of activated cells seen on 25 kPa hydrogels with 30% being αSMA^+^ and 30% being αSMA^i^ (Figure S1b–d). Interestingly, further increases in hydrogel stiffness led to a slight reduction in myofibroblast activation similar to the reduction seen in YAP N/C (Figure S1c,d). This saturation and slight reduction in activation with stiffness may be due to the saturation of the mechanotransduction machinery at high stiffness [31, 32, 33]. Thus, FN coated ECM stiffness of 25 kPa promotes maximal transient myofibroblast activation in vCAFs which correlates with maximal activation of stiffness‐dependent mechanotransduction pathways.

**FIGURE 1:**
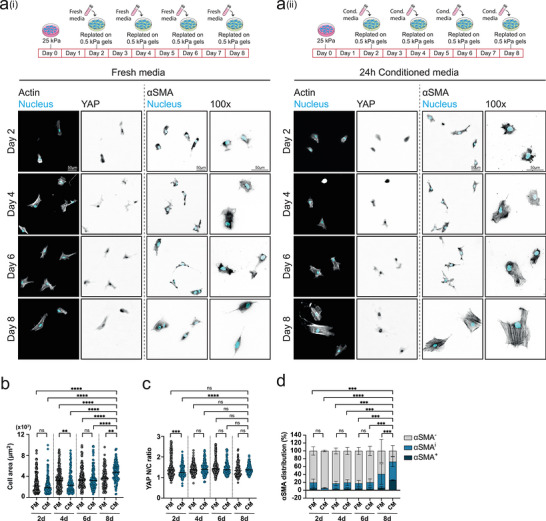
Persistent myofibroblast activation requires prolonged exposure to stiff ECM and stiffness‐dependent secreted factors. (a(i)) Schematic representation of the experimental workflow to test for stiffness mediated persistent myofibroblast activation in the presence of fresh media. Representative cropped 20x images of vCAFs replated on 0.5 kPa hydrogels with fresh media following culturing on 25 kPa hydrogels for different periods of time, showing actin (gray), nucleus (cyan), YAP (gray) and 20x and 100x images of aSMA (gray). (a(ii)) Schematic representation of the experimental workflow to test for stiffness mediated persistent myofibroblast activation in the presence of 24 h conditioned media. Representative cropped 20x images of vCAFs replated on 0.5 kPa hydrogels with 24 h conditioned media following culturing on 25 kPa hydrogels for different periods of time, showing actin (gray), nucleus (cyan), YAP (gray) and 20x and 100x images of aSMA (gray). (b,c) Quantification of cell area (b) and YAP N/C ratio (c) from vCAFs replated on 0.5 kPa hydrogels with fresh media and 24 h conditioned media following culturing on 25 kPa hydrogels for different periods of time (n =170–188 (2d), 147–170 (4d), 157–181 (6), 97–110 (8d) cells for fresh media and n = 159–191 (2d), 161–170 (4d), 148–150 (6d), 151–176 (8d) cells for conditioned media from 3 experimental repeats). Kruskal–Wallis test, ^**^ *P* ≤ 0.01, ^***^ *P* ≤ 0.001, ^****^ *P* <0,0001, ns = not significant. Bars represent median values. (d) αSMA distribution quantified from vCAFs replated on 0.5 kPa hydrogels with fresh media and 24 h conditioned media following culturing on 25 kPa hydrogels for different periods of time. (n =173 (2d), 300 (4d), 200 (6d), 382 (8d) cells for fresh media and n =321 (2d), 282 (4d), 178 (6d), 191 (8d) cells for conditioned media from 3 experimental repeats). Statistical tests done between αSMA^+^ cells. Ordinary one‐way ANOVA, ^***^ *P* ≤ 0.001, ^****^ *P* <0,0001. Error bars represent mean with SEM. αSMA^+^: activated, αSMA^i^: intermediately activated, αSMA^−^: not activated cells.

We next sought to identify conditions where ECM‐stiffness mediated transient myofibroblast activation transformed to persistent myofibroblast activation defined as cells remaining active upon being re‐plated on soft ECM after being cultured on stiff ECM. To do so, we assessed the ability of cells to be active on 0.5 kPa FN‐coated hydrogels 20 h post replating, following culturing on 25 kPa hydrogels for either 2days (d), 4d, 6d, or 8d (Figure 1a(i)). However, we found that even though YAP N/C was high, cells had reduced cell spread areas and αSMA^+^ cells (Figure 1b–d). Since some previous studies on persistent activation use in situ softening of hydrogels, it suggested to us a potential role for secreted components in persistent activation which was lost when cells were re‐plated on 0.5 kPa hydrogels with fresh media [21, 28, 34]. Thus, we modified our protocol by replating cells on 0.5 kPa hydrogels with conditioned media collected from the last 24 h of culturing on 25 kPa hydrogels (Figure 1a(ii)). Cells replated on 0.5 kPa hydrogels following 2d, 4d, or 6d of culturing on 25 kPa hydrogels with their corresponding 24 h conditioned media showed increasing YAP N/C and cell spread areas while very few were αSMA^+^ cells, indicating no persistent myofibroblast activation (Figure 1b–d). However, cells cultured for 8d on 25 kPa hydrogels and then replated on 0.5 kPa hydrogels with 24 h conditioned media (d7‐8 media) were significantly more spread with high YAP N/C. Quantification of αSMA revealed that these culturing condition resulted in nearly 25% cells being αSMA^+^ and 50% being αSMA^i^ (Figure 1d), similar to cells transiently activated on 25 kPa hydrogels, thus indicating that these were persistently activated myofibroblasts. Additionally, we confirmed true persistent activation by fixing cells cultured on 0.5 kPa hydrogels for 8d following 8d culture on 25 kPa hydrogels and found that they also remained active showing high number of αSMA^+^ cells with large cell spread areas and YAP N/C ratios (Figure S1e–h).

To check if this requirement of extended culturing on stiff ECM along with culturing‐derived secreted components to obtain persistently activated myofibroblasts was specific for vCAF cell lines, we repeated the protocol with telomerase‐immortalized normal fibroblasts (TIFs) (Figure S2a–e) and primary human lung fibroblasts (Figure S2f–j) and found similar requirements of extended culturing that resulted in persistent myofibroblast activation.

We next tested if ECM‐stiffness derived secreted components was sufficient for persistent myofibroblast activation or if extended culturing on stiff ECM was also required. Cells cultured for 8d on 0.5 kPa hydrogels were replated on 0.5 kPa hydrogels with 24 h conditioned media from cells cultured on 25 kPa hydrogels for 8d. Conversely, cells cultured for 8d on 25 kPa hydrogels were re‐plated on 0.5 kPa hydrogels with 24 h conditioned media from cells cultured on 0.5 kPa hydrogels for 8d. In both cases there were very few αSMA^+^ and αSMA^i^ cells on 0.5KPa hydrogels (Figure S2k). Additionally, cells cultured for 6d on 25 kPa hydrogels and then re‐plated on 0.5 kPa hydrogels with 24 h conditioned media from cells trained on 25 kPa hydrogels for 8d also had very few αSMA^+^ cells but with an increase in αSMA^i^ cells (Figure S2k).

These results show that ECM‐stiffness mediated persistent myofibroblast activation requires both extended exposure to stiff ECM and the presence of ECM stiffness dependent secreted components.

### 2.2 Nuclear Lamina and Chromatin are Remodelled during Prolonged Exposure to High ECM Stiffness

Biophysical cues from the ECM can actively modulate nuclear architecture and chromatin organization to regulate the epigenome of cells undergoing fate switching and differentiation [22, 24]. Building on previous work on ECM‐stiffness mediated regulation of chromatin architecture during myofibroblast activation, we first set out to confirm the role of chromatin modification in persistent myofibroblast activation of vCAFs by inhibiting histone modifications during extended culturing on stiff ECM. Briefly, vCAFs cultured on 25 kPa FN‐coated hydrogels for 8d were treated with Trichostatin A (TSA) to inhibit Class I and Class II histone deacetylases (HDACs) or Garcinol to block histone acetyltransferases (HATs) [35, 36]. To minimize potential cytotoxicity, TSA, Garcinol or DMSO (vehicle controls) were added on day 6 of culturing for 24 h and replaced with fresh media on day 7. Cells were then replated onto 0.5 kPa hydrogels with 24 h conditioned media as done previously and assessed for αSMA^+^ cells, cell spread area and YAP N/C 20 h post replating (Figure S3a). Imaging and quantification revealed that while TSA treatment did not affect cell spreading, Garcinol treated cells had mildly reduced cell spread areas compared to DMSO‐treated controls (Figure S3a,b). Further, TSA and Garcinol treatments significantly reduced YAP N/C (Figure S3c) while only TSA reduced the number of αSMA^+^ myofibroblasts (Figure S3d,e) compared to DMSO‐treated controls. To test if this effect on persistent activation was due to TSA and Garcinol affecting transient activation, cells cultured on 25 kPa hydrogels were treated with TSA, Garcinol or DMSO and assessed for activation 20 h post treatment on day 7. Here we found that while TSA treatment had a mild effect on YAP N/C, neither TSA nor Garcinol affected the percentage of αSMA^+^ cells (Figure S3f–j). This suggests that an increase or decrease in chromatin acetylation does not affect transient activation during prolonged exposure to a stiff ECM. However, inhibiting HDACs specifically reduces ECM‐stiffness mediated persistent activation in vCAFs.

**FIGURE 2:**
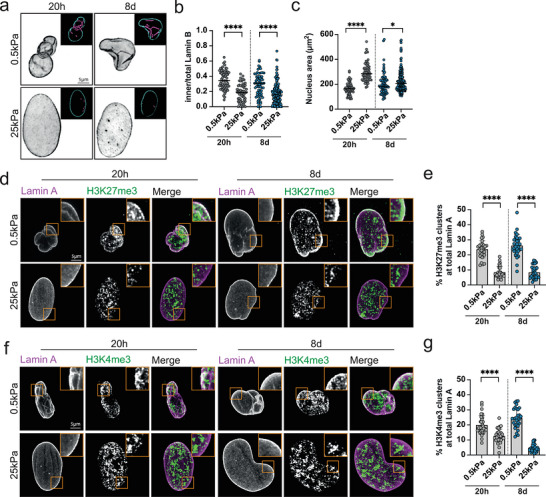
Nuclear lamina and chromatin are remodelled during ECM stiffness mediated persistent myofibroblast activation. (a) Representative cropped deconvolved 60x Ti2 images showing Lamin B (gray) from vCAFs cultured on 0.5 and 25 kPa hydrogels for 20 h and 8d. Inset shows inner (magenta) and outer (cyan) Lamin B segmentations. (b,c) Quantification of inner/total lamin B area (b) and Nuclear area (c) from vCAFs cultured on 0.5 and 25 kPa hydrogels for 20 h and 8d. (n = 64–66 (20 h 0.5 kPa), 57 (20 h 25 kPa), 73‐75 (8d 0.5 kPa), 158 (8d 25 kPa) cells from 3 experimental repeats) Mann Whitney test, ^*^ *P* ≤ 0.05, ^****^ *P* <0,0001. Bars represent median values. (d) Representative cropped deconvolved 60x confocal images showing Lamin A and H3K27me3 from vCAFs cultured on 0.5 and 25 kPa hydrogels for 20 h and 8d. Merge shows Lamin A (magenta) and H3K27me3 (green). Images are background subtracted. (e) Quantification of percentage of H3K27me3 clusters at total Lamin A. Total lamin A is the sum of inner and outer lamin A segmentations (n = 30–31 cells for each condition from 3 experimental repeats). Mann Whitney test, ^****^ *P* <0,0001. Error bars represent mean with SD. (f) Representative cropped deconvolved 60x confocal images showing Lamin A and H3K4me3 from vCAFs cultured on 0.5 and 25 kPa hydrogels for 20 h and 8d. Merge shows Lamin A (magenta) and H3K4me3 (green). Images are background subtracted. (g) Quantification of percentage of H3K4me3 clusters at total lamin A. Total lamin A is the sum of inner and outer lamin A segmentations. (n = 30 cells for each condition from 3 experimental repeats). Mann Whitney test, ^****^ *P* <0,0001. Error bars represent mean with SD.

Changes in ECM stiffness have been shown to influence chromatin organization through multiple pathways, including nucleoskeletal force transmission, cytoskeletal tension, and mechanosensitive transcriptional programmes [21, 37, 38]. To investigate this link, we next quantified changes in nuclear morphology and nuclear lamins during extended culturing on stiff and soft ECM. vCAFs on 25 kPa FN coated hydrogels were fixed and stained for Lamin B after 20 h or 8d of extended culturing and compared with cells cultured on 0.5 kPa hydrogels fixed at the same timepoints. Deconvolved immunofluorescent widefield images revealed extensive wrinkling of the nuclear lamina in cells cultured on 0.5 kPa hydrogels compared to cells on 25 kPa hydrogels at both timepoints (Figure 2a). Consistent with these observations, quantification of inner and total lamin folds showed that after both 20 h and 8d on the hydrogels, cells on 0.5 kPa hydrogels had more than double the number of lamin folds compared to cells on 25 kPa hydrogels (Figure 2b). Further, smoothened lamina on 25 kPa hydrogels coincided with a larger nuclear area compared to on 0.5 kPa hydrogels (Figure 2c). Because of the differences in lamin organization between cells on 0.5 and 25 kPa hydrogels, we next quantified changes in lamin folds during the entire extended culturing period (2d, 4d, 6d and 8d) for cells on the 25 kPa hydrogels and observed small changes in lamin folds across the days with cells mostly maintaining a relatively smooth lamina during the entire culturing period (Figure S4a,b). Similarly, we found no changes in nuclear area from 2 to 6d with a mild increase on 8d (Figure S4c), suggesting that the nuclear lamina remains morphologically smooth during extended culturing on stiff ECM for persistent myofibroblast activation.

The physical interaction between specific regions of the chromatin and the nuclear lamina can regulate chromatin accessibility for transcription of several mechanoresponsive genes [25, 26, 27, 39]. Since our data above shows that chromatin modifications play a critical role in ECM‐stiffness mediated persistent activation, but that the nuclear lamina is itself morphologically stable, we next hypothesized that the localization of specific regions of the chromatin relative to the nuclear lamina may change during extended culturing. To test this, we spatially mapped the localization of H3 lysine 27 trimethylation (H3K27me3) and H3 lysine 4 trimethylation (H3K4me3), known markers for heterochromatin and euchromatin respectively, relative to the nuclear lamina, thus enabling assessment of changes in chromatin organization at the nuclear lamina. First, we fixed and stained vCAFs plated for either 20 h or 8d on 0.5 and 25 kPa hydrogels for H3K27me3 and H3K4me3 and stained the nuclear lamina with an antibody for Lamin A. We switched to Lamin A here instead of Lamin B because of species differences with the co‐stained histone antibodies. We verified that lamin folds marked by Lamin A antibody were quantitatively similar as those marked by Lamin B under these conditions through co‐staining experiments which showed average Manders' correlation coefficients between 0.88 and 0.98 (Figure S4d,e). Histone clusters and the nuclear lamina were segmented and the percentage of H3K27me3 or H3K4me3 clusters in contact with the nuclear lamina was quantified and compared (see methods). vCAFs on 0.5 kPa hydrogels showed a relatively high fraction of H3K27me3 clusters along the lamin folds compared to cells on 25 kPa hydrogels, both after 20 h and 8d of extended culturing (Figure 2d,e). Similarly, vCAFs on 0.5 kPa hydrogels also showed a higher fraction of H3K4me3 clusters along the lamin folds compared to cells on 25 kPa hydrogels at both time points (Figure 2f,g). To further resolve the timing of this shift on 25 kPa hydrogels during extended culturing, we repeated this analysis at different timepoints and found that the fraction of H3K27me3 clusters in close proximity of the lamin folds remained relatively low (compared to cells on 0.5 kPa hydrogels) and unchanged during the 8d (Figure S4f,g) while there was a significant change in the fraction of H3K4me3 clusters away from the nuclear lamina after 20 h of culturing and remained unchanged over the remaining 8d (Figure S4h,i).

Taken together, these results show that ECM stiffness‐mediated persistent myofibroblast activation requires chromatin remodelling that coincides with chromatin redistribution around the nuclear lamina during prolonged exposure on stiff ECM.

### 2.3 ECM‐Stiffness Dependent Persistent Myofibroblast Activation Requires Activation of β1 Family Integrins during Prolonged Exposure to Stiff ECM

**FIGURE 3:**
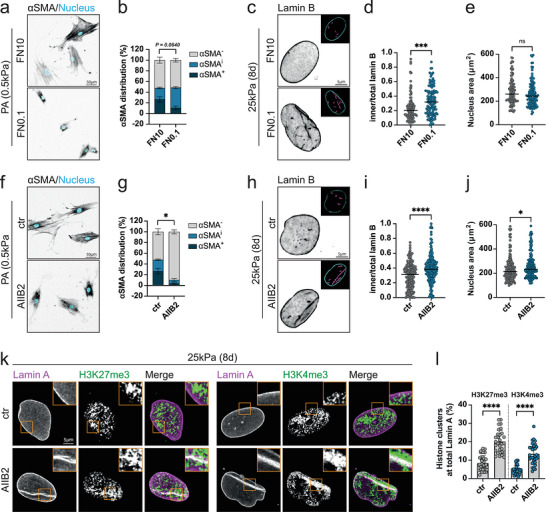
ECM‐stiffness dependent persistent myofibroblast activation requires activation of β1 family integrins. (a,b) Representative cropped 20x images of vCAFs replated on 0.5 kPa hydrogels following 8d culture on 25 kPa hydrogels coated with either 10 or 0.1 mg/mL of FN, showing nucleus (cyan) and αSMA (gray) (a) and quantification of αSMA distribution (b). (n = 240 (FN10), 207 (FN0.1) cells from 3 experimental repeats). Statistical tests done between αSMA+ cells. Welch's t test. Error bars represent mean with SEM. (c) Representative cropped deconvolved 60x Ti2 images showing Lamin B (gray) from vCAFs cultured on 25 kPa hydrogels coated with either 10 or 0.1 mg/mL of FN on 8d. Inset shows inner (magenta) and outer (cyan) Lamin B segmentations. (d,e) Quantification of inner/total lamin B area (d) and nuclear area (e) from vCAFs cultured on 25 kPa hydrogels coated with either 10 or 0.1 mg/mL of FN on 8d. (n = 95 (FN10), 114 (FN01) cells from 3 experimental repeats). Mann Whitney test, ^***^ *P* ≤ 0.001, ns = not significant. Bars represent median values. (f,g) Representative cropped 20x images of vCAFs replated on 0.5 kPa hydrogels following treatment with AIIB2 during 8d culture on 25 kPa hydrogels and control, showing nucleus (cyan) and αSMA (gray) (f) and quantification of αSMA distribution (g). (n = 240 (ctr), 259 (AIIB2) cells from 3 experimental repeats). Statistical tests done between αSMA+ cells. Welch's *t* test, ^*^ *P* ≤ 0.05. Error bars represent mean with SEM. Note: ctr data same as FN10 in Figure 3b. (h) Representative cropped deconvolved 60x Ti2 images showing Lamin B (gray) from vCAFs cultured on 25 kPa hydrogels for 8d following treatment with AIIB2 and control. Inset shows inner (magenta) and outer (cyan) Lamin B segmentations. (i, j) Quantification of inner/total lamin B area (i) and nuclear area (j) from vCAFs cultured on 25 kPa hydrogels for 8d following treatment with AIIB2 and control. (n = 146 (ctr), 172 (AIIB2) cells from 3 experimental repeats). Mann Whitney test, ^*^ *P* ≤ 0.05, ^****^ *P* <0,0001. Bars represent median values. (k) Representative cropped deconvolved 60x confocal images showing Lamin A and H3K27me3 (left) and H3K4me3 (right) from vCAFs cultured on 25 kPa for 8d following treatment with AIIB2 and control. Merge shows Lamin A (magenta) and H3K27me3/H3K4me3 (green). Images are background subtracted. (l) Quantification of percentage of H3K27me3 and H3K4me3 clusters at total Lamin A. Total lamin A is the sum of inner and outer lamin A segmentations. (n = 30‐31 cells for each condition from 3 experimental repeats). Mann Whitney test, ^****^ *P* <0,0001. Error bars represent mean with SD. Note: 8d ctr data same as 8d data in Figure 2e and 2g. αSMA^+^: activated, αSMA^i^: intermediately activated, αSMA^−^: not activated cells. PA: persistent activation.

Our data above shows that conditions that lead to persistent myofibroblast activation correlate with maximal activation of mechanotransduction pathways as measured by YAP N/C during extended culturing on stiff ECM compared to cells on soft hydrogels. Since activation of these stiffness‐dependent pathways requires binding and activation of specific integrins, we investigated this next. To first test if integrin binding and a threshold of activation was required, we reduced FN density on 25KPa hydrogels from 10 to 0.1 µg/mL. vCAFs were cultured for 8 days on 0.1 µg/ml FN coated 25 kPa hydrogels and re‐plated on 10 µg/mL FN coated 0.5 kPa hydrogels with their 24 h conditioned media for 20 h, just as before. Under the low FN condition, cells replated on 0.5 kPa hydrogels showed diffused αSMA staining, indicating loss of persistent myofibroblast activation (Figure 3a). This was confirmed by quantification which showed that vCAFs cultured on 0.1 µg/mL FN coated 25 kPa hydrogels lost a large fraction of αSMA^+^ myofibroblast cells on 0.5 kPa hydrogels (Figure 3b) with lower cell spread area and a reduction in YAP N/C compared to cells replated from 10 µg/mL FN coated 25 KPa hydrogels (Figure S5a–c). Since low FN densities reduced persistent activation on soft substrates, we tested if low FN densities also affected transient activation on stiff substrates. Cells cultured on 0.1 µg/mL FN coated 25 kPa hydrogels for 20 h had reduced cell spread area and YAP N/C as expected but this had only a mild and statistically insignificant effect on the number of αSMA^+^ myofibroblast cells compared to 10 µg/mL controls (Figure S5d–h). While reducing ligand concentrations can affect multiple cell behaviors such as cell spreading, these results indicate that this does not affect short term transient activation but affects ECM‐stiffness mediated persistent activation. Further, this suggests that a threshold level of integrin‐ECM ligand binding and activation during extended culturing on stiff ECM is required for this process. We next quantified changes in lamin organisation and nuclear morphology and found that cells cultured on FN0.1‐coated 25 kPa hydrogels showed extensive wrinkling of the nuclear lamina at 8d (Figure 3c,d), with no significant difference in projected nuclear area compared to cells on FN10 (Figure 3e). Quantification of nuclear volume revealed that cells on FN0.1 had greater nuclear volumes than cells on FN10 (Figure S5i,j), suggesting that wrinkling reflects out‐of‐plane nuclear deformation rather than in‐plane stretching, and that nuclear wrinkling correlates more strongly with 3D nuclear geometry than with projected area alone.

Fibroblasts attach to FN primarily through β1 family of integrins heterodimers with α5β1 being the most abundantly expressed in fibroblasts [40, 41]. To investigate the role of β1 integrins in ECM‐stiffness mediated persistent activation, we first performed a functional validation test for the β1 integrin blocking antibody AIIB2. Cells treated with AIIB2 for 20 h displayed reduced cell spread area and a marked loss of FAs containing α5 integrins as demonstrated by reduced SNAKA51 staining on FN coated glass, indicating a loss of α5β1 integrins (Figure S6a–c). Following this, we then treated vCAFs with AIIB2 for the first 3 days of culturing on 25KPa hydrogels before washing it out and culturing the cells for a total of 8d just as before. Cells were then replated on 0.5KPa hydrogels with their 24 h conditioned media and measured for persistent myofibroblast activation, cell spread area and YAP N/C 20 h post plating. This data showed that blocking β1integrins during culturing on stiff ECM was sufficient to significantly reduce αSMA^+^ cells on 0.5KPa hydrogels (Figure 3f,g) along with a significant reduction in YAP N/C and a surprising increase in cell area (Figure S6d–f). This increase in cell spread area following β1 blocking may be due to compensatory effects resulting in the upregulation or activation of alternate integrins which can promote cell spreading without restoring cellular contractility. We next tested if the effect of β1 activation on persistent activation was due to its role in transient activation during culturing on stiff substrates and found that vCAFs treated with AIIB2 for 20 h on 25 kPa hydrogels had reduced YAP N/C ratios, cell spread area and αSMA^+^ cells (Figure S6g–k), indicating that β1 engagement is required for both transient and persistent activation.

To further investigate the specificity of β1 integrins binding to FN, we tested if vCAFs can acquire persistent activation following 8d culture on collagen I or vitronectin coated stiff ECMs. Fibroblasts primarily bind to collagen I through α11β1, α2β1 and α1β1 and vitronectin through αvβ3 and αvβ5. Just as before, cells were replated on FN coated 0.5 kPa hydrogels with their 24 h conditioned media following 8d culture on collagen I, vitronectin or FN coated 25 kPa hydrogels. 20 h post re‐plating, cells cultured on collagen I coated 25 kPa hydrogels displayed high cell spread areas and YAP N/C with a large number of αSMA+ cells, similar to FN controls (Figure S7f–j). However, in comparison, cells cultured on vitronectin coated 25 kPa hydrogels had lower cell spread areas, YAP N/C and αSMA+ cells (Figure S7f–j), indicating that persistent vCAF activation is ECM composition dependent and on FN is primarily mediated by β1 integrins.

To next test if blocking of β1 integrins can affect nuclear morphology and lamin organization, we measured changes in these properties during extended culturing on 25KPa hydrogels in the presence of AIIB2 and compared it to untreated controls after 20 h or 8d of culturing. After 20 h of culturing, blocking of β1 integrins did not alter nuclear lamin folds (Figure S7a,b) but reduced nuclear area compared to untreated controls (Figure S7c). However, at the end of the extended culturing period, i.e after 8d, blocking of β1 integrins led to significant nuclear lamina wrinkling compared to untreated controls (Figure 3h,i) with concurrent increase in nuclear area (Figure 3j).

Since the folding of the nuclear lamin upon blocking of β1 integrins during extended culturing on stiff ECM was reminiscent of lamin folding during extended culturing on soft ECM, both of which lead to loss of persistent myofibroblast activation, we next asked if localization of heterochromatin and euchromatin at the lamin was also altered in absence of β1 integrin activation. vCAFs cultured on 25KPa hydrogels and treated with AIIB2 were fixed and stained for H3K27me3 or H3K4me3 and co‐stained with Lamin A at 20 h or 8d of culturing. After 20 h of culturing, AIIB2 treated cells did not show any difference in localization of H3K27me3 or H3K4me3 clusters along the lamin (Figure S7d,e). In contrast, after 8d of extended culturing, inhibition of β1 integrin activation with AIIB2 led to a significant increase in fraction of H3K27me3 and H3K4me3 clusters at the lamin folds to levels similar to cells cultured on soft ECM (Figure 3k,l). These results show that ECM binding and activation of β1 integrin above a certain threshold during prolonged exposure to stiff FN coated ECM is required for persistent myofibroblast activation and nuclear lamina smoothening that alters its physical association with chromatin.

### 2.4 mDia2 is Required during Prolonged Exposure to Stiff ECM for ECM‐Stiffness Mediated Persistent Activation and Chromatin Organization Independent of Nuclear Lamina‐Actin Coupling

**FIGURE 4:**
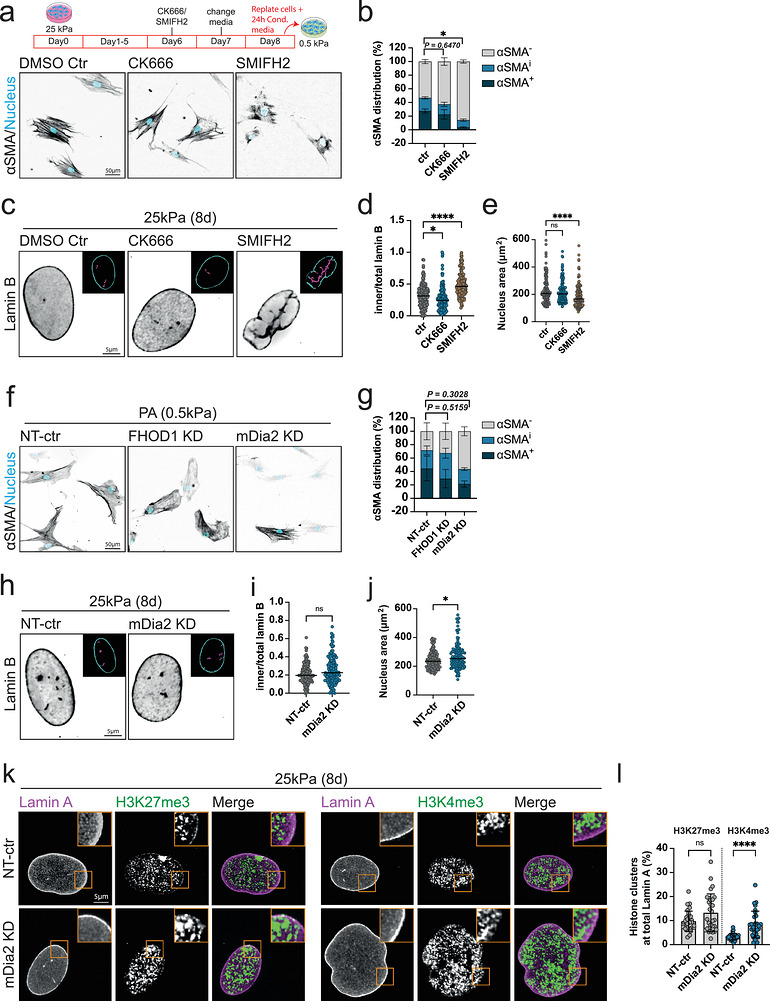
mDia2 is required for ECM‐stiffness mediated persistent activation and chromatin organization independent of nuclear lamina‐actin coupling. (a,b) Schematic representation of the experimental workflow. Representative cropped 20x images of vCAFs replated on 0.5 kPa hydrogels following treatment with CK666, SMIFH2 or DMSO during 8d culture on 25 kPa hydrogels, showing nucleus (cyan) and αSMA (gray) (a) and quantification of αSMA distribution (b). (n = 234 (DMSO ctr), 196 (CK666), 226 (SMIFh2) cells from 3 experimental repeats). Statistical tests done between αSMA+ cells. Ordinary one‐way ANOVA, ^*^ *P* ≤ 0.05. Error bars represent mean with SEM. (c) Representative cropped deconvolved 60x Ti2 images showing Lamin B (gray) from CK666, SMIFh2 or DMSO treated vCAFs cultured on 25 kPa hydrogels for 8d. Inset shows inner (magenta) and outer (cyan) Lamin B segmentations. (d,e) Quantification of inner/total lamin B area (d) and nuclear area (e) from CK666, SMIFh2 or DMSO treated vCAFs cultured on 25 kPa hydrogels for 8d. (n = 148 (DMSO ctr), 139 (CK666), 148 (SMIFh2) cells from 3 experimental repeats). Kruskal Wallis test, ^*^ *P* ≤ 0.05, ^****^ *P* <0,0001, ns = not significant. Bars represent median values. (f,g) Representative cropped 20x images of NT‐ctr, FHOD1 KD and mDia2 KD vCAFs replated on 0.5 kPa hydrogels following 8d culture on 25 kPa hydrogels, showing nucleus (cyan) and αSMA (gray) (f) and quantification of αSMA distribution (g). (n = 145 (NT‐ctr), 99 (FHOD1 KD), 282 (mDia2 KD) cells from 3 experimental repeats). Statistical tests done between αSMA+ cells. Ordinary one‐way ANOVA. Error bars represent mean with SEM. (h) Representative cropped deconvolved 60x Ti2 images showing Lamin B (gray) from NT‐ctr and mDia2 KD cells cultured on 25 kPa hydrogels for 8d. Inset shows inner (magenta) and outer (cyan) Lamin B segmentations. (i,j) Quantification of inner/total lamin B area (i) and nuclear area (j) from NT‐ctr and mDia2 KD cells cultured on 25 kPa hydrogels for 8d. (n = 128 (NT‐ctr), 128 (mDia2 KD) cells from 3 experimental repeats). Mann Whitney test, ^*^ *P* ≤ 0.05, ns = not significant. Bars represent median values. (k) Representative cropped deconvolved 60x confocal images showing Lamin A and H3K27me3 (left) and H3K4me3 (right) from NT‐ctr and mDia2 KD cells cultured on 25 kPa for 8d. Merge shows Lamin A (magenta) and H3K27me3/H3K4me3 (green). Images are background subtracted. (l) Quantification of percentage of H3K27me3 and H3K4me3 clusters at total Lamin A. Total lamin A is the sum of inner and outer lamin A segmentations. (n = 30 cells for each condition from 3 experimental repeats). Mann Whitney test, ^****^ *P* <0,0001, ns = not significant. Error bars represent mean with SD. αSMA^+^: activated, αSMA^i^: intermediately activated, αSMA^−^: not activated cells. PA: persistent activation.

Since our data above suggests a physical link between ECM dependent integrin activation and changes in chromatin architecture via remodeling of the nuclear lamin during prolonged exposure on stiff ECM for persistent myofibroblast activation, we next aimed to elucidate this physical link. Several previous studies have implicated dynamic spatiotemporal regulation of actin assembly by formins and the Arp2/3 complex in coupling chromatin organization or the nuclear lamina to integrin‐based focal adhesions [42, 43]. To first test if ECM stiffness mediated persistent myofibroblast activation requires assembly of actin filaments during extended culturing on stiff ECM, we treated vCAFs with either CK‐666 or SMIFH2 to inhibit the Arp2/3 complex and the formin family respectively during the extended culturing. Briefly, cells being cultured on FN‐coated 25 kPa hydrogels were treated with either CK666, SMIFH2 or DMSO on day 6 for 24 h and then washed out and replaced with fresh media on day 7 prior to being replated on FN‐coated 0.5 kPa hydrogels on day 8 (Figure 4a). Fixing and staining for αSMA, YAP and actin 20 h post re‐plating showed that only inhibition of formins significantly reduced the percentage of αSMA^+^ myofibroblasts (Figure 4a,b), YAP N/C and cell spread area (Figure S8a–c) while inhibiting the Arp2/3 complex had no effect. We then tested whether SMIFH2 affects transient activation of vCAFs on soft substrates. Cells cultured on 25 kPa hydrogels treated with SMIFH2 for 20 h on day 7 showed reduced YAP N/C and αSMA^+^ myofibroblasts while cell spread area stayed the same, thus suggesting that this effect of SMIFH2 is through its effect on transient activation (Figure S8d–h). To further investigate whether the effect of formin inhibition was mediated by changes in nuclear morphology and lamin architecture during extended culturing on stiff ECM, we fixed and stained cells on d8 of culturing on 25 kPa for the nuclear lamins. Cells treated with SMIFH2 showed extensive wrinkling of the nuclear lamina with smaller nuclear area compared to controls and CK666 treated cells (Figure 4c–e). Thus, ECM‐stiffness mediated persistent myofibroblast activation requires formin activity during prolonged exposure on stiff ECM.

We next aimed to identify the specific formin required for ECM‐stiffness mediated persistent myofibroblast activation. We focused on two formin family members, Fhod1 and Diaph3/mDia2 due to their known role in regulating actin assembly in and around the nucleus that may influence nuclear morphology and chromatin organization. FHOD1 has been shown to tether the nucleus to the actin cytoskeleton via the LINC complex, enabling force transmission between the cytoskeleton and the nucleus [44, 45]. mDia2, which has established roles in regulating actin assembly, microtubule dynamics, and mitochondrial positioning in the cytoplasm, has additionally been shown to shuttle into the nucleus where it assembles short actin filaments that control intranuclear chromatin movements and influence gene expression [4, 46, 47]. SiRNAs targeting either FHOD1 or mDia2 and a non‐targeting (NT) control were used to downregulate their expression in vCAFs (Figure S12) during extended culturing on 25KPa substrates prior to replating on 0.5KPa hydrogels. Interestingly, while FHOD1 and mDia2 KD cells still spread on 0.5KPa substrates after 8d of culturing on stiff ECM, downregulation of mDia2 expression led to a greater reduction in αSMA^+^ and αSMA^i^ cells (Figure 4f,g). While there was no effect on cell spread area compared to NT controls, we found that there was a significant reduction in YAP N/C in mDia2 KD cells replated on 0.5 kPa hydrogels as well (Figure S8i–k). Again, this effect could be due to mDia2 affecting transient activation during the prolonged exposure on stiff ECM. To test for this, mDia2 KD cells plated on 25 kPa hydrogels for 20 h were measured for activation and we found no changes in cell spread, YAP N/C ratio or percentage of αSMA^+^ cells compared to NT controls (Figure S9a–e). Thus, mDia2 is not required for transient activation but only for persistent activation on soft substrates after prolonged exposure on stiff ECM.

We next investigated if downregulation of mDia2 also affected nuclear morphology, lamin architecture and chromatin organization during extended culturing on stiff ECM. To do so, we fixed mDia2 KD and NT control cells after 20 h or 8d of culturing on 25KPa hydrogels and first stained for Lamin B to analyse lamin wrinkling and nuclear morphology. Interestingly, unlike cells on soft ECM or during integrin blocking on stiff ECM, downregulation of mDia2 had no significant effect on lamin folds and cells had mostly a smooth nuclear lamina at 20 h (Figure S9f,g) or day 8 (Figure 4h,i) with a mild increase in nuclear area on d8 (Figure 4j) but not at 20 h (Figure S9h). Consistent with this, we found no significant change in F‐actin architecture surrounding the nucleus in mDia2 KD cells compared to NT controls (Figure S9i,j). We followed this analysis with analysis of heterochromatin and euchromatin localization using H3K27me3 and H3K4me3 staining respectively just as before. This revealed that while at 20 h there were no changes in H3K27me3 and H3K4me3 localization at the lamins in mDia2 KD cells (Figure S9k,l), after 8d of culturing on 25 kPa, mDia2 KD cells had a significant increase in H3K4me3 clusters at the lamins compared to NT controls while there was no change in H3K27me3 distribution at the lamina (Figure 4k,l). In the absence of extensive lamin folding and changes in F‐actin architecture surrounding the nucleus in mDia2 KD cells during extended culturing on 25 kPa hydrogels, we hypothesized that changes in euchromatin localization at the lamins may relate to the role of mDia2 in regulating actin in the nucleus. To test this, we quantified the average axial distribution of phalloidin stained F‐actin within the nuclear volume of NT control and mDia2 KD cells after 20 h of culturing on 25 kPa hydrogels (Figure S10a). This analysis revealed that while NT control cells showed a clear peak in F‐actin distribution within the nuclear volume near the centre of the nucleus, this peak was significantly attenuated in mDia2 KD cells suggesting reduction in F‐actin levels within the inner regions of the nucleus in the absence of mDia2 (Figure S10a, b). To further confirm this, we transfected mDia2 KD cells with nuclear actin chromobody (nAC‐GFP) [48]. Consistent with its role in the nucleus, mDia2 KD cells showed poor localization of the chromobody to the nucleus compared to the NT controls (Figure S10c,d), suggesting that mDia2 KD cells have altered nuclear actin organization.

Taken together, these results show that the formin mDia2 is required during prolonged exposure to stiff ECM for persistent myofibroblast activation, acting independent of nuclear lamin‐actin coupling through its potential role within the nucleus in regulating chromatin reorganization. Additionally, our results with the formin inhibitor suggests that additional formins, besides mDia2 (and FHOD1), coordinates nuclear lamin‐actin coupling downstream of integrin activation to promote ECM stiffness dependent persistent myofibroblast activation.

### 2.5 Histone Deacetylation Acts Downstream of Integrin and mDia2 Signalling to Mediate Persistent Myofibroblast Activation

**FIGURE 5:**
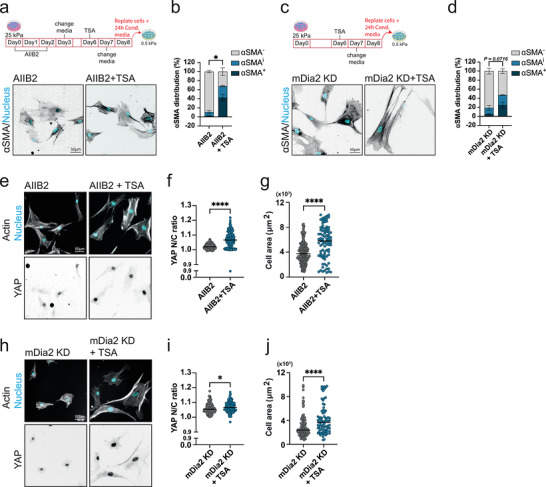
Histone deacetylation acts downstream of integrin and mDia2 signalling to drive persistent myofibroblast activation. (a,b) Schematic representation of the experimental workflow. Representative cropped 20x images of vCAFs replated on 0.5 kPa hydrogels following treatment with AIIB2 and AIIB2 + TSA during 8d culture on 25 kPa hydrogels, showing nucleus (cyan) and αSMA (gray) (a) and quantification of αSMA distribution (b). (n = 125 (AIIB2), 117 (AIIB2 + TSA) cells from 3 experimental repeats). Statistical tests done between αSMA^+^ cells. Welch's t test, ^*^ *P* ≤ 0.05. Error bars represent mean with SEM. (c,d) Schematic representation of the experimental workflow. Representative cropped 20x images of mDia2 KD vCAFs replated on 0.5 kPa hydrogels following treatment with TSA during 8d culture on 25 kPa hydrogels, showing nucleus (cyan) and αSMA (gray) (c) and quantification of αSMA distribution (d). (n = 178 (mDia2), 171 (mDia2 KD + TSA) cells from 3 experimental repeats). Statistical tests done between αSMA+ cells. Welch's *t* test. Error bars represent mean with SEM. (e) Representative cropped 20x images of vCAFs replated on 0.5 kPa hydrogels following treatment with AIIB2 and AIIB2 + TSA during 8d culture on 25 kPa hydrogels, showing actin (gray), nucleus (cyan) and YAP (gray). (f,g) Quantification of YAP N/C (f) and cell area (g) from vCAFs replated on 0.5 kPa hydrogels following treatment with AIIB2 and AIIB2 + TSA during 8d culture on 25 kPa hydrogels. (n = 186–233 (AIIB2), 85–186 (AIIB2 + TSA) cells from 3 experimental repeats). Mann Whitney test, ^****^ *P* <0,0001. Bars represent median values. (h) Representative cropped 20x images mDia2 KD vCAFs replated on 0.5 kPa hydrogels following treatment with TSA during 8d culture on 25 kPa hydrogels, showing actin (gray), nucleus (cyan) and YAP (gray). (i,j) Quantification of YAP N/C (i) and cell area (j) from mDia2 KD vCAFs replated on 0.5 kPa hydrogels following treatment with TSA during 8d culture on 25 kPa hydrogels. (n = 105–137 (mDia2 KD), 81–119 (mDia2 KD + TSA) cells from 3 experimental repeats). Mann Whitney test, ^*^ *P* ≤ 0.05, ^****^ *P* <0,0001. Bars represent median values. αSMA^+^: activated, αSMA^i^: intermediately activated, αSMA^−^: not activated cells.

Our results so far indicate that activation of β1 integrins and the formin mDia2 during prolonged exposure to FN enriched stiff ECM is required for persistent myofibroblast activation on soft ECM. Additionally, we find that activation of these pathways coincides with chromatin reorganization near the nuclear lamina, driven through distinct pathways— β1 integrins via changes in lamin architecture and mDia2 potentially via regulation of nuclear F‐actin. We next hypothesized that these ECM‐stiffness‐dependent pathways that lead to physical changes in chromatin organization ultimately need to affect chromatin modification and function. To test this, we decided to alter HDAC activity downstream of β1 integrin and mDia2 inhibition and investigate its effect on persistent myofibroblast activation (Figure 5). Briefly, β1 integrins blocked or mDia2 KD vCAFs were cultured on 25 kPa hydrogels and treated with TSA on day 6 of extended culturing. The media was washed out and replaced with fresh media on day 7 and replated on 0.5 kPa hydrogels on day 8 and analysed for persistent myofibroblast activation 20 h post re‐plating. Analysis of αSMA^+^ cells under these conditions showed that while blocking of β1 integrins or knocking down of mDia2 during extended culturing on stiff ECM led to a reduction in αSMA^+^ cells on 0.5 kPa hydrogels, the addition of TSA to either β1 integrins blocked or mDia2 KD cells during the extended culturing rescued these effects and resulted in a significant increase in αSMA^+^ cells (Figure 5a–d). Consistent with this, the addition of TSA also resulted in increases in YAP N/C and cell spread area, thus indicating a complete restoration of persistent myofibroblast activation under these conditions (Figure 5e–j). This suggested to us that specific histone modifications are downstream of ECM‐stiffness mediated β1 integrin and mDia2 activation.

We next investigated if this effect of TSA on rescuing the effects of β1 integrin blocking or mDia2 downregulation was dependent on chromatin organization and its association with the nuclear lamina. We thus stained for heterochromatin and euchromatin using H3K27me3 and H3K4me3 antibodies respectively in β1 integrin‐blocked or mDia2 KD vCAFs cells cultured for 8d on 25 kPa hydrogels and treated with TSA (Figure S11). Analysis of H3K27me3 and H3K4me3 clusters around the nuclear lamina showed that while TSA treatment led to a reduction in H3K27me3 clusters at the lamin in β1 integrins blocked vCAFs after 8 days of extended culturing, it had no effect on the localization of H3K4me3 in β1 integrins blocked or on mDia2 KD cells (Figure S11a–f), suggesting that the rescue effects on persistent myofibroblast activation was independent of significant changes in the physical association between chromatin and the nuclear lamins. Taken together, these results suggest that chromatin modifications including histone deacetylation act downstream of β1 integrin and mDia2 mediated mechanotransduction pathways to drive ECM stiffness dependent persistent myofibroblast activation, even in the absence of chromatin repositioning.

## 3 Discussion

The switch in plasticity of activation from transient to persistent states in fibroblasts is a critical regulator of fibrosis. Here, we identify pathways through which CAFs undergo this switch upon prolonged exposure to stiff ECM. Our findings show that CAFs acquire a persistent myofibroblast phenotype through ECM‐dependent mechanotransduction pathways that downstream regulate chromatin state. We find that this requires both continuous exposure to a stiff ECM and the accumulation of stiffness‐induced secreted factors over that time‐period. CAFs have been shown to secrete a number of autocrine signals to promote and sustain myofibroblast activation. Based on prior studies, possible factors could be TGF‐β1, which is stored in the ECM as a latent complex and is mechanically released by myofibroblast contractile force in a stiffness‐dependent manner — a process that does not occur on compliant substrates and requires integrin‐mediated force transmission [49, 50]. This stiffness‐gated TGF‐β1 release creates an autocrine reinforcement loop that would be expected to accumulate over the 8‐day culture period and would be present in conditioned media, consistent with our observations. Notably, β1 integrin blocking has been shown to inhibit latent TGF‐β1 stress activation [49], providing a potential mechanistic link between our integrin results and the requirement of conditioned media. Lysophosphatidic acid (LPA), produced locally by autotaxin secreted from activated fibroblasts, is another strong candidate since LPA has been shown to sustain myofibroblast contractility via RhoA/ROCK signalling in an autocrine fashion [51, 52]. Identifying the relevant factors would require conditioned media proteomics combined with candidate depletion experiments, which are studies we are planning to do in the future.

Mechanistically, we uncover two distinct but potentially related pathways linking the ECM to the chromatin, one that is β1 integrin‐lamin organization dependent and the other that is the formin mDia2 dependent but independent of changes in lamin organization, likely through its role in assembling actin in the nucleus. Both pathways downstream converge on altering chromatin‐lamin interactions. Importantly, persistent activation occurs even in the absence of large‐scale chromatin repositioning and ECM‐dependent mechanotransduction pathways, highlighting chromatin modifications as critical downstream effectors underlying this activation switch. Consistent with this, our results show that while integrin blocking affects transient activation and affects nuclear lamina similar to fibroblasts that are not transiently activated on soft substrates, this is distinct from the effects of TSA and mDia2 knockdown. These perturbations effect persistent activation on soft substrates without affecting transient activation on stiff substrates and altering the nuclear lamina. This suggests to us that changes in chromatin organization and modifications are ultimately downstream of the mechanotransduction process that drives persistent activation. Broadly, our results also suggest that epigenetic program in CAFs driven by a self‐sustaining mechanochemical feedback loop may underlie the establishment of a pro‐metastatic tumor niche.

Our study complements previous work on persistent fibroblast activation in fibrosis and extends these concepts to the TME, providing potential mechanisms by which myCAFs acquire a stable contractile phenotype that supports metastasis [5, 18, 53, 54]. Importantly, by demonstrating that both CAFs and immortalized normal fibroblasts (TIFs) and primary fibroblasts can transition to a persistently activated state under prolonged stiff ECM exposure, we show that this switch may not merely be a pathological response but an inherent feature of fibroblast biology. Interestingly, this phenomena of mechanical memory have been extended to epithelial cells which show ECM stiffness dependent memory during cell migration [55]. Taken together, this suggests that stiffness dependent changes in the epigenome may be a more fundamental phenomena underlying several tissue and cell types.

Prior studies have established that persistent activation relies on engaging mechanotransduction pathways to activate epigenetic reprograming, with altered histone acetylation and chromatin modifications acting as central drivers of fibroblast “mechanical memory” [18, 21, 23, 34, 56, 57, 58]. Here, we add that these epigenetic outcomes can be initiated through fundamentally physical pathways: integrin–mediated remodeling of the nuclear lamina that alters lamin–chromatin associations, and the nuclear function of the formin mDia2 in organizing actin and influencing chromatin positioning. Further, these epigenetic changes persist despite cytoskeletal and mechanotransducive remodeling during trypsinization and enzymatic dissociation while replating, supporting a chromatin‐centric mechanism of memory. Together with our finding suggesting that histone deacetylation lies downstream of these inputs, our results highlight that multiple mechanochemical routes converge on chromatin modification to enforce persistent activation. This convergence underscores chromatin as the critical integrative node of fibroblast plasticity and raises new questions about how distinct nuclear pathways might be differentially engaged in fibrosis vs. cancer.

Hydrogels used in the study were coated with fibronectin, the primary ECM ligand for β1 integrin heterodimers in fibroblasts and a major upregulated component of fibrotic and tumour stroma [59], making it physiologically appropriate substrate for investigating stiffness‐dependent integrin‐mediated mechanotransduction in CAFs. Mechanistically, we identify FN binding β1 integrin activation downstream of ECM stiffness as the initiating event in the mechanochemical cascade that drives the switch from transient to persistent activation in fibroblasts. A key outstanding question here is which downstream effectors distinguish short‐term vs. long‐term integrin signaling in this context [60]. Candidates may include canonical focal adhesion regulators, as well as mechanosensitive transcriptional cofactors like YAP/TAZ and MRTF/SRF, whose dynamics likely differ between acute and sustained engagement [61, 62]. Sustained force transmission to the nuclear lamina may additionally require reinforcement through alternative pathways, including other integrin subtypes or stretch‐activated ion channels such as Piezo1 or TRPV4, whose contribution in this process remains unexplored and undefined [63]. Notably, αv integrins, which also bind FN, play established roles in latent TGF‐β activation and SMAD‐dependent transcriptional programs, suggesting that crosstalk between integrin subtypes and growth factor pathways could provide an additional layer of regulation distinguishing transient from persistent fibroblast activation [64, 65]. Within the TME, where the ECM is heterogeneous in composition, density, and architecture, integrin crosstalk and context‐specific recruitment of adhesion regulators are likely to add further complexity to how nuclear structure and chromatin organization are modulated [66]. Dissecting how distinct ECM components and integrin subtypes cooperate or compete to stabilize persistent activation will be critical for understanding stromal plasticity in both cancer and fibrosis.

Several studies have identified key molecular components that link ECM mechanics to the nucleus and influence chromatin organization. These include focal adhesion proteins such as integrins and talin, cytoskeletal elements like actin and microtubules, and nuclear envelope components such as the LINC complex and lamins [67]. It is now well established that mechanical forces can be transmitted from the cell surface to the nucleus and even to the chromatin itself, through both lamin‐dependent and ‐independent pathways. Recent work has also highlighted a role for actin nucleators, including formins and the Arp2/3 complex, in regulating nuclear organization both by linking the cytoskeleton to the nucleus and by acting directly within the nuclear compartment [42, 67]. In our system, we find that ECM stiffness–dependent persistent myofibroblast activation requires formin activity but is independent of Arp2/3. We further identify mDia2 as a critical formin that mediates this process, acting within the nucleus to alter euchromatin organization, likely through regulation of nuclear actin polymerization [68]. This aligns with previous studies implicating mDia2 in nuclear actin dynamics and chromatin regulation [69, 70]. Alternate targets could be transcriptional co activators of SMA such as MRTF‐A and serum response factor that were found to have reduced nuclear transport and reduced expressions respectively in mDia knockdown cells [71]. However, as with integrins, our data suggest that other formins also contribute to ECM‐to‐nucleus coupling, and identifying these factors remains an important area for future work. We also recognize that our current identification of mDia2's nuclear role is preliminary and will require further validation using live cell imaging of the chromobody in mDia2 KD cells to capture dynamics of nuclear actin assembly in response to stiffness probes and functional mDia2 mutants. Nevertheless, these findings raise the intriguing possibility that ECM mechanics regulate the epigenome through formin‐dependent pathways—mechanisms that remain largely unexplored.

Studies have shown that chromatin–lamina associations play a key role in transcriptional repression and are responsive to mechanical cues from the extracellular matrix. Lamina‐associated domains (LADs), which often bear repressive histone marks such as H3K9me2, are spatially enriched at the nuclear periphery [72]. While these marks alone are associated with gene silencing, their ability to repress transcription appears to require physical tethering to the nuclear lamina. Notably, repression at the nuclear periphery is not limited to heterochromatic loci—evidence suggests that even regions marked by euchromatic modifications can become transcriptionally silent when relocated to LADs, emphasizing the role of spatial positioning as a dominant regulator of gene activity [73]. Disruption of this organization, even with intact chromatin marks, has been shown to impair silencing and interfere with cell state transitions. In addition to mechanical remodeling of the lamina, nuclear actin dynamics may further influence chromatin positioning, as monomeric actin interacts with chromatin modifiers and can modulate chromatin accessibility [74]. Based on our results, we speculate that persistent myofibroblast activation may involve stabilization of LAD–lamina interactions through both mechanical and actin‐dependent mechanisms. These could include changes in lamin architecture, local chromatin tension, or shifts in the availability of nuclear actin that influence the physical tethering of chromatin. Together, these processes may reinforce transcriptional repression at specific loci and contribute to the establishment of an epigenetic memory in fibroblasts exposed to sustained mechanical input. Future studies using ATAC‐seq or CUT&RUN‐based profiling will be critical to map changes in chromatin accessibility and lamin‐associated domains in this context.

Epigenetic regulation through histone acetylation and DNA methylation has recently emerged as a key driver of mechanical memory, with chromatin condensation states shown to be more sensitive than cellular morphological responses [75, 76]. In the context of fibrosis, HATs activity enhances chromatin accessibility, promoting pro‐fibrotic gene expression whereas HDACs reduce chromatin accessibility and contribute to the silencing of pro‐fibrotic loci [77]. Our data are consistent with this framework, though the effects of TSA reveal a context‐dependent role for HDAC activity. In normally activating cells, TSA reduced persistent activation, consistent with evidence that global chromatin decondensation can paradoxically impair specific transcriptional programs by disrupting compartmentalization of active and repressed loci [21]. In cells where β1 integrin signaling or nuclear actin organization has been disrupted, however, we propose that the correct chromatin state fails to be established during extended stiff ECM culture, with HDAC activity becoming aberrantly elevated, consistent with evidence that integrin engagement directly modulates HDAC localization and activity [78, 79, 80]. This excessive deacetylation epigenetically silences fibroblast activation loci and TSA treatment restores acetylation at these sites, rescuing persistent activation. Importantly, this rescue occurs without restoring lamin‐chromatin associations, indicating that TSA acts at the level of chromatin modification rather than physical chromatin repositioning. Together, these findings position histone deacetylation as a downstream effector of both the β1 integrin and mDia2 pathways, and demonstrate that persistent myofibroblast activation is encoded in a specific chromatin modification state rather than in gross nuclear architecture.

Together, our findings reveal a multilayered mechanochemical framework by which fibroblasts integrate sustained mechanical cues into persistent changes in nuclear architecture and chromatin state. While we begin to delineate how specific integrins and nuclear actin regulators shape this process, much remains to be uncovered about how these pathways operate in the full complexity of the tumor microenvironment. In particular, understanding how diverse ECM components, dynamic tissue architecture, and cell–cell interactions modulate fibroblast persistence will be essential to define the range and stability of CAF phenotypes in vivo. Moreover, identifying which chromatin regions are functionally rewired during this process, and how these changes interact with transcription factor networks or lineage programs, remains an open challenge. Addressing these questions will be key to linking fibroblast epigenetic memory to broader tissue‐level behaviors, including matrix remodeling, immune modulation, and cancer progression.

## 4 Methods

### 4.1 Cell Culture

Vulval cancer associated fibroblasts (vCAFs) were a gift from Dr. Chris Madsen [81, 82, 83] and telomerase‐immortalized fibroblasts (TIFs) were a gift from Dr. Sandeep Gopal (Lund University, SE) [84, 85]. Human primary lung fibroblasts were obtained from BioIVT (237‐090). The cell lines have not been authenticated by the authors but have been previously well‐characterized. The cell lines were chosen based on these previous characterization studies which show that vCAFs and TIFs are good model cell lines for CAFs and normal fibroblasts respectively [81, 82, 83, 84, 85]. RRIDs for these cell lines are not available. All vCAF experiments were performed between passages 5 and 25. Cells were maintained on plastic tissue culture dishes and passaged every 3 days. To confirm that prior culture on plastic did not confer mechanical memory, cells seeded directly onto 0.5 kPa hydrogels following standard plastic culture displayed no persistent αSMA+ activation.

vCAFs were cultured in DMEM (Gibco, 15140122) supplemented with 10% FBS (Gibco, 10270106), Insulin‐Transferrin‐Selenium (Gibco, 41400045) and penicillin/Streptomycin (Gibco, 15140122) at 37°C and 5% CO2. TIFs were cultured in DMEM (Gibco, 15140122) supplemented with 10% FBS (Gibco, 10270106) and penicillin/Streptomycin (Gibco, 15140122) at 37°C and 5% CO2. Gels used in our experimental setups were Matrigen easy coat Softwell polyacrylamide hydrogels. Gels were coated with 10 µg/ml fibronectin (Sigma‐Aldrich, F0895‐2 mg), collagen (Cell systems, 5153‐1KIT) or vitronectin (Fisher Scientific, A14700) for 30 min at 37°C and blocked with 2% BSA (Sigma–Aldrich, A7906) in PBS for 30 min at 37°C prior to plating cells. Cells assessed for persistent activation were fixed and stained 20 h post re‐plating on 0.5 kPa gels following 8d culturing on 25 kPa gels. Cells assessed for transient activation were fixed and stained at 20 h or 7d of culturing on 25 kPa gels. While cells have not been tested for mycoplasma contamination by the authors, no aberrant changes in cell morphology or proliferation were noted during the course of the study. As such, the absence of mycoplasma testing is not expected to compromise the experimental findings.

### 4.2 Immunostaining

For YAP, lamin and histone immunostaining, cells were fixed with 4% paraformaldehyde (Thermo scientific, 28906) diluted in PBS for 15 min at 37°C. Cell membrane was permeabilized with 0.2% Triton‐X (Alfa Aesar, A16046) in PBS for 5 min followed by a blocking step with 3% BSA in PBS for 1 h. Cells were incubated with primary antibodies (1:400) in 1% BSA in PBS for 2 h at RT. Primary antibodies used were: YAP monoclonal IgG2a antibody (Santa Cruz biotechnology, sc‐101199), Lamin B1 polyclonal antibody (Proteintech, 12987‐1‐AP), Lamin A 4A58 (Santa Cruz biotechnology, sc‐71481), H3K4me3 Monoclonal antibody (Invitrogen, MA5‐11199), H3K27me3 Polyclonal antibody (Invitrogen, PA5‐31817). Cells were washed with 0.1% Tween in PBS 3x 5 min and incubated with secondary antibodies and phalloidin (1:400) in 1% BSA in PBS for 1 h at RT. Secondary antibodies used were: Alexa Flour goat anti‐mouse IgG 647 nm (Invitrogen, A21235), Alexa Flour goat anti‐rabbit IgG 647 nm (Invitrogen, A21244), Alexa Flour goat anti‐mouse IgG 488 nm (Invitrogen, A11001), Alexa Flour goat anti‐mouse IgG 568 nm (Invitrogen, A11031), phalloidin 488 (Invitrogen, A12380), phalloidin 568 (Invitrogen, A11011). Cells were washed with PBS and incubated with nuclear dye Hoechst 350 (1:4,000) in PBS for 15 min at RT (Thermo Fisher Scientific, 33342). Cells were washed with 0.1% Tween in PBS 2x 5 min and PBS 2x 5 min. Gels were kept hydrated in PBS and imaged.

For αSMA and α5 immunostaining, cells were fixed with 4% paraformaldehyde (Thermo scientific, 28906) diluted in cytoskeleton buffer (CB) (10 mm MES pH 6.1, 3 mm MgCl2, 135 mm KCl, 2 mm EGTA) for 20 min at 37°C. Cells were permeabilized with 0.2% Triton‐X (Alfa Aesar, A16046) in CB for 5 min. Formaldehyde was quenched with 0.1 m Glycine (Sigma, 50046‐250G) in CB for 10 min and washed with TBS 3x 5 min. Cells were blocked with 4% BSA in TBS with 0.5% tween (BTT) for 1 h and incubated with anti‐alpha SMA ACTA2 antibody (Merck, A2547) (1:400) or anti‐integrin alpha 5 antibody (SNAKA51) (Merck, MABT201) (1:400) in BTT for 2 h at RT. Cells were washed with 0.1% Tween in PBS 3x 5 min and incubated with secondary antibodies and phalloidin (1:400) in BTT in PBS for 1 h at RT. Secondary antibodies used were the same as used above. Cells were washed with PBS and incubated with nuclear dye Hoechst 350 (1:4,000) in PBS for 15 min at RT (Thermo Fisher Scientific, 33342) and washed with 0.1% Tween in PBS 2x 5 min and PBS 2x 5 min. Gels were kept hydrated in PBS and imaged.

### 4.3 Integrin Blocking Assays

To block β1 integrins, cells were pre‐incubated in cell culture media with 3 µg/ml Anti‐Integrin Beta1 (CD29), clone AIIB2 antibody (Merck, MABT409) for 20 min at RT. Cells were then plated onto gels with media containing the same concentrations of integrin blocking antibody. Integrin blocking antibody were replenished for the first 3 days of culture. Media was replaced with fresh media on the fourth day of culture.

### 4.4 Actin Polymerization Inhibition Assays

Arp2/3 complex or Formin mediated actin polymerization was inhibited with 100 µm of CK‐666 (Merck, 182515) or 20 µm SMIFH2 (Merck, S4826) respectively on day 6 of culture. Cells were treated for 24 h and replaced with fresh media on day 7.

### 4.5 Histone Deacetylation and Acetyltransferases Inhibition Assays

Histone deacetylation was inhibited with 200 nm trichostatin A (Tocris Bioscience, 1406) and 5 µm garcinol (Stemcell technologies, 72452) was used to inhibit histone acetyltransferases on day 6 of culture. Cells were treated for 24 h and replaced with fresh media on day 7.

### 4.6 siRNA and DNA Transfection

Cells were seeded in 6‐well plates, and standard lipofectamine 3000 protocol was followed. Briefly, 50pM non‐targeting (Dharmacon, D‐001810‐10‐05), FHOD1 targeting (Dharmacon, L‐013709‐01‐0005) or mDia2 targeting (Dharmacon, L‐018997‐00‐0005) siRNA was incubated in serum free media with Lipofectamine 3000 transfection reagent for 15 min prior to adding to cells. 2.5 µg of Nuclear Actin Chromobody TagGFP plasmid (Proteintech, acg‐­n) in serum free media with P3000 reagent was incubated with lipofectamine 3000 reagent for 15 min prior to adding to cells. Cells were incubated for 48 h before seeding on gels.

### 4.7 Western Blotting

Cells were washed twice with ice cold PBS and lysed with RIPA buffer (Thermo fisher Scientific, 89901) + protease inhibitors (Thermo fisher Scientific, A32961) on ice for 10 min. Cells were scraped, and lysates were centrifuged at 12 000 rpm for 10 min at 4°C. Supernatant was collected and denatured with 4x Laemmli sample buffer (Biorad,1610747) + β‐mercaptoethanol and heated to 95°C for 10 min. Protein separation by gel electrophoresis was done using Tris‐Glycine gels (Invitrogen, XP04202BOX). Biorad *Trans*‐blot kit (Biorad, 1704274) was used to transfer proteins to PVDF membranes. Membranes were blocked with 3% BSA in TBS with 1% tween for 1 h at RT. Membranes were incubated with a‐Tubulin (Invitrogen, 14‐4502‐80) and DIAPH3 (Proteintech, 14342‐1‐AP) or FHOD1 (Santa Cruz biotechnology, sc‐365437) primary antibodies in 3% BSA overnight. Membranes were washed 3x with TBS with 1% tween for 5 min and incubated with Starbright Blue 700 Goat Anti‐Mouse IgG (Biorad, 12004158) or Starbright Blue 520 Goat Anti‐Rabbit IgG (Biorad, 12005869) for 1 h at RT. Membranes were washed 3x with TBS with 1% tween for 5 min and imaged with a ChemiDoc MP system (Biorad).

### 4.8 Wide‐Field Fluorescence Imaging

A Nikon Ti2‐E widefield fluorescence microscope was used to acquire images of cells stained for SMA, YAP, actin and Lamin B. Images were acquired with 640‐, 561‐, 488‐ and 405‐nm LED light source (Lumencor SpectraX light engine). Images were acquired using a Nikon DS‐Qi2 CMOS camera. SMA, YAP and actin were imaged with an APO 20 × 0.75 N.A. objective. SMA was acquired as single plane images and YAP and actin was acquired as z‐stacks. Lamin B and actin were imaged with Nikon CFI SR Plan Apo IR 60XAC WI / 1.27NA objective as z stacks and Lamin B images were deconvolved using the Richardson and Lucy deconvolution algorithm available in the NIS elements software and used to measure Lamin B folds.

### 4.9 Confocal Imaging

A Nikon Confocal A1RHD microscope was used to acquire images of cells stained for H3K4me3 and H3K27me3 histone methylations with Lamin A and for nuclear volume measurements. Images were acquired using 640‐, 561‐, 488‐ and 405‐nm laser lines equipped with GaAsP PMTs. Plan Apochromal Λ 60x 1.42 NA oil objective was used with the Nikon Nyquist acquisition. Z‐stacks with a step size of 0.21 µm was used to capture the entire nuclear volume. Images were deconvolved using the Nikon Automatic deconvolution algorithm available in the NIS elements software.

### 4.10 Quantification and Statistical Analysis

#### 4.10.1 Cell Area, Yap Ratio and αSMA Quantification

Cell area and nuclear to cytoplasmic YAP ratio quantifications were performed on 20x widefield fluorescence images using FIJI and cell profiler. Briefly, image stacks from each channel were processed in FIJI. Z‐stack images were projected using the “Z Project” function with maximum intensity projection to generate 2D representations. CellProfiler was then employed to segment nuclei and whole cells using DAPI and Actin channels, respectively. Based on the segmentation results, cellular morphological features such as cell area were quantified. The cytoplasmic region was defined by subtracting the segmented nuclear mask from the whole‐cell mask. YAP ratio was determined by dividing the mean fluorescence intensity of the YAP antibody in the nuclear region by that of the cytoplasmic region in the YAP channel. A size cut‐off of 600‐10 000 µm2 was used for cell area and GraphPad Prism's outlier analysis was used to filter YAP ratio data.

α‐smooth muscle actin (αSMA) organization was quantified using a custom Python‐based image analysis pipeline based on a previously developed pipeline [86]. Raw αSMA images were background‐corrected using large‐scale Gaussian filtering for segmenting cells while minimally processed images were used for αSMA measurements. Cells were segmented from αSMA signal using thresholding and morphological refinement, and αSMA organization was quantified within a peripheral region of interest using a structure‐tensor–based coherence metric, where values range from isotropic (0) to highly aligned fibrous structures (1). Per‐cell coherence values were computed from αSMA‐positive pixels and used to classify cells into non‐activated, intermediate, or activated states based on predefined thresholds. Per‐cell measurements were aggregated across images within each experiment, and the percentage of activated cells per experiment was used as the primary outcome for statistical analysis. Experiments were treated as independent biological replicates.

#### 4.10.2 Nuclear Volume, Actin Fibre Alignment and Actin Distribution Quantification

Nuclear volume quantifications were done using the ImageJ plugin 3D object counter on 60x confocal images. Actin fibre alignment was quantified using the ImageJ plugin FibrilTool on 60x Ti2 actin images. Axial distribution of nuclear actin was quantified from deconvolved 60x confocal images by drawing line scans across xy orthogonal views of actin in regions of the nucleus in Fiji.

#### 4.10.3 Lamin A and Lamin B Colocalization Quantification

Colocalization of Lamin A and Lamin B was analyzed using the ImageJ plugin JACoP available in BIOP (Bioimaging and Optics Platform) to calculate Manders' correlation coefficients.

#### 4.10.4 Lamin B Segmentation and Quantification in Widefield Images

Nuclei segmentation was performed using an image processing pipeline. Maximum intensity projections were generated from the z‐stacks, and the resulting 2D images were smoothed using a Gaussian filter (σ = 5). Otsu thresholding was applied to binarize nuclear regions, and objects smaller than 500 pixels in area were excluded to remove noise and small artifacts.

Lamin fold segmentation was carried out on deconvolved images. Images were normalized by dividing by the background median intensity, estimated via sigma‐clipped statistics. Localized curvature structures were enhanced using a Laplacian of Gaussian filter (σ = 1.0), followed by Gaussian smoothing (σ = 5.0) to suppress residual noise. A preliminary segmentation was obtained by thresholding based on global image statistics. To further refine this, an inner nuclear mask was generated using median filtering and Otsu thresholding, enabling more accurate estimation of local background intensity. This refinement was used to improve the specificity of fold segmentation. Finally, segments smaller than 50 pixels were removed, and the resulting mask was used to quantify Lamin B fold area per nucleus.

#### 4.10.5 Lamin B Segmentation in Confocal Images

Lamin B segmentation in confocal images was performed using a nucleus‐localized workflow. Nuclei were segmented as described above. For each nucleus, a cropped region was extracted from the deconvolved lamin channel, and the local background statistics were estimated via sigma‐clipped mean and standard deviation within the overlapping nuclear region. Pixels exceeding the local background by one standard deviation were retained, and contrast‐rich features were enhanced using a Laplacian of Gaussian filter (σ = 3). Segmentation was performed using adaptive thresholding [87], and small objects below 100 pixels were discarded. To improve coverage and suppress residual noise, a secondary pass was performed using median filtering, LoG (σ = 2), and Otsu thresholding, followed by size filtering (minimum 10 pixels). The union of both masks was used to quantify Lamin B fold area and intensity within each cropped region.

#### 4.10.6 Histone Localization

Histone puncta were identified using a local‐maxima detection pipeline. For each nucleus, a cropped region of the histone channel was extracted, and local background statistics were estimated via sigma‐clipped mean and standard deviation. Candidate puncta were detected as local intensity maxima within nuclear boundaries using a 5 × 5 sliding window. Each spot was fitted with a 2D Gaussian curvature model to refine its position and validate shape consistency. Optimization was performed using bounded quasi‐Newton with 10 iterations and parameter constraints on curvature terms. Spots overlapping image borders were excluded, and the fraction of detected histone puncta spatially overlapping the segmented lamin fold regions was computed per nucleus.

### 4.11 Statistical Analysis

All data was analyzed using GraphPad Prism version 9 (GraphPad Software, Boston, Massachusetts USA). Statistical details can be found in the figure legends.

## Author Contributions

Conceptualization was carried out by S.P. and V.S. Data curation was performed by S.P. and V.S. Formal analysis was conducted by S.P., O.A., Z.F., and V.S. Funding acquisition was secured by V.S. Investigation was performed by S.P. Methodology was developed by S.P. and V.S. Project administration was handled by V.S. Resources were provided by P.N. and V.S. Software was developed by O.A., Z.F., P.N., and V.S. Visualization was carried out by S.P., O.A., and V.S. The original draft was written by S.P. and V.S. Review and editing were performed by S.P., O.A., Z.F., P.N., and V.S. Supervision was provided by V.S.

## Conflicts of Interest

The authors declare no conflicts of interest.

## Supporting information




**Supporting File**: advs75066‐sup‐0001‐SuppMat.pdf.

## Data Availability

The data that support the findings of this study are available from the corresponding author upon reasonable request.
