# Supplementary material for: ECM‐Stiffness Mediated Persistent Fibroblast Activation Requires Integrin and Formin Dependent Chromatin Remodeling
Source: Adv Sci (Weinh). 2026 Mar 31;13(34):e17631. doi: 10.1002/advs.202517631 (PMC13285145; doi:10.1002/advs.202517631)
Supplement: Supplementary file 1 — Supporting File: advs75066‐sup‐0001‐SuppMat.pdf. [file ADVS-13-e17631-s001.pdf]

Figure S1

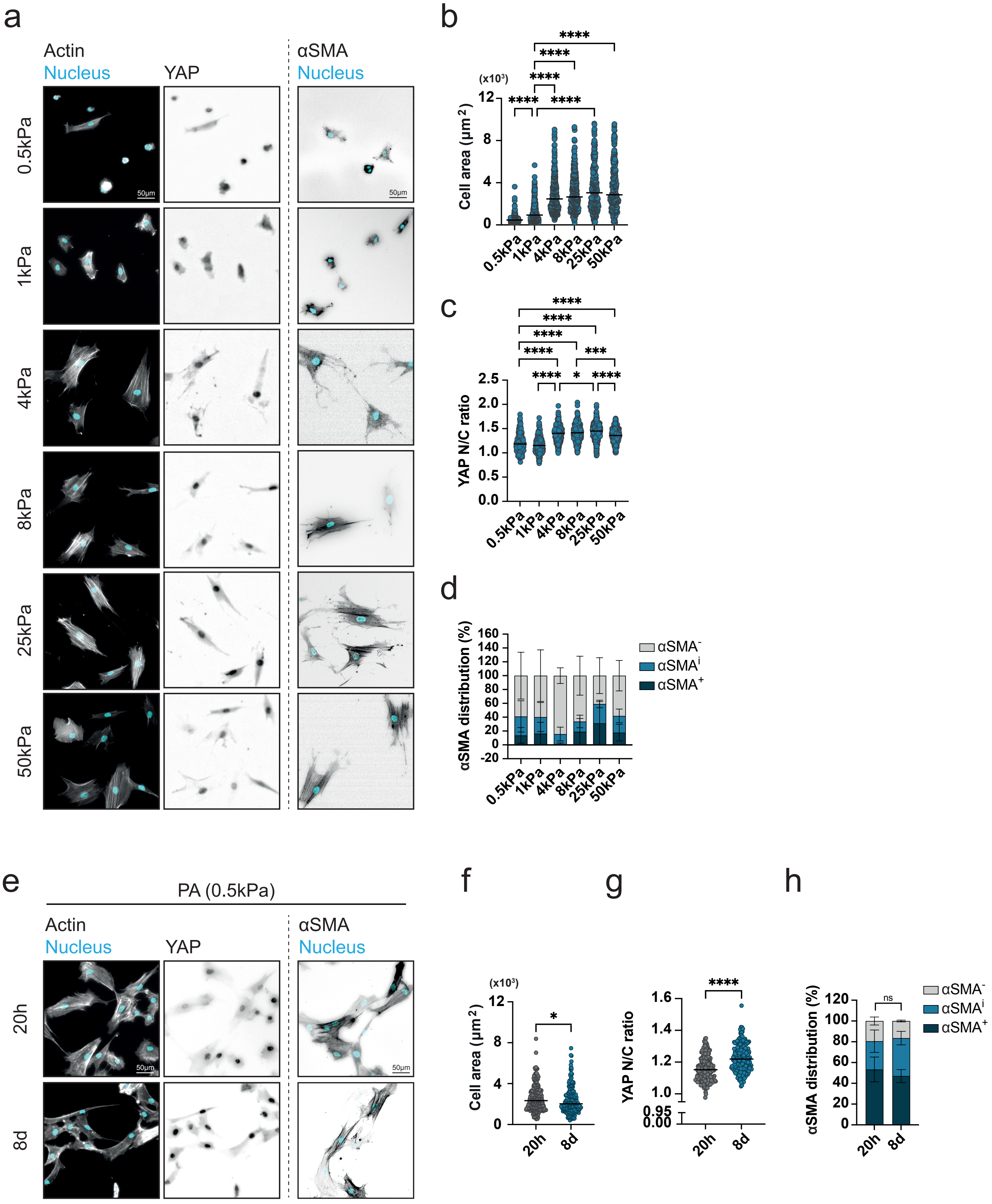

a
Actin
Nucleus
YAP
αSMA
Nucleus
0.5kPa
1kPa
4kPa
8kPa
25kPa
50kPa
50µm
b
(x10³)
Cell area (µm²)
0
4
8
12
****
0.5kPa
1kPa
4kPa
8kPa
25kPa
50kPa
c
YAP N/C ratio
0.0
0.5
1.0
1.5
2.0
2.5
****
***
*
d
αSMA distribution (%)
-20
0
20
40
60
80
100
120
140
160
αSMA⁻
αSMAⁱ
αSMA⁺
e
PA (0.5kPa)
20h
8d
f
g
h
1.6
1.4
1.2
1.0
0.95
0.00
ns

Figure S1

(a) Representative cropped 20x images of vCAFs cultured on hydrogels of different stiffnesses for 20h, showing actin (gray), nucleus (cyan), YAP (fgray) and αSMA (gray). **(b,c)** Quantification of cell area **(b)** and YAP N/C ratio **(c)** from vCAFs cultured on hydrogels of different stiffnesses for 20h. (n = 196-224 (0.5kPa), 237-286 (1kPa), 318-375 (5kPa), 312-362 (8kPa), 285-354 (25kPa), 275-328 (50kPa) cells from 2 experimental repeats). Kruskal-Wallis test, *P ≤ 0.05, ***P ≤ 0.001, ****P <0,0001. Bars represent median values. **(d)** Quantification of αSMA distribution in vCAFs cultured on hydrogels of different stiffnesses for 20h. (n = 76 (0.5kPa), 109 (1kPa), 104 (5kPa), 93 (8kPa), 68 (25kPa), 91 (50kPa) from 2 experimental repeats). **(e)** Representative cropped 20x images of vCAFs re-plated on 0.5kPa hydrogels for 20h or 8d following 8d culture on 25kPa hydrogels, showing actin (gray), nucleus (cyan), YAP (fgray) and αSMA (gray). **(f,g)** Quantification of cell area **(f)** and YAP N/C ratio **(g)** from vCAFs re-plated on 0.5kPa hydrogels for 20h or 8d following 8d culture on 25kPa hydrogels. (n= 210 (20h), 208 (8d) cells from 3 experimental repeats). Mann-Whitney test, *P ≤ 0.05, ****P <0,0001. Bars represent median values. **(h)** Quantification of αSMA distribution in vCAFs re-plated on 0.5kPa hydrogels for 20h or 8d following 8d culture on 25kPa hydrogels. (n= 130 (20h), 97 (8d) from 3 experimental repeats) Statistical tests done between αSMA^{+} cells. Welch's t test, ns = not significant. Error bars represent mean with SEM. αSMA^{+}: activated, αSMA^{i}: intermediately activated, αSMA^{-}: not activated cells. PA : persistent activation

Figure S2

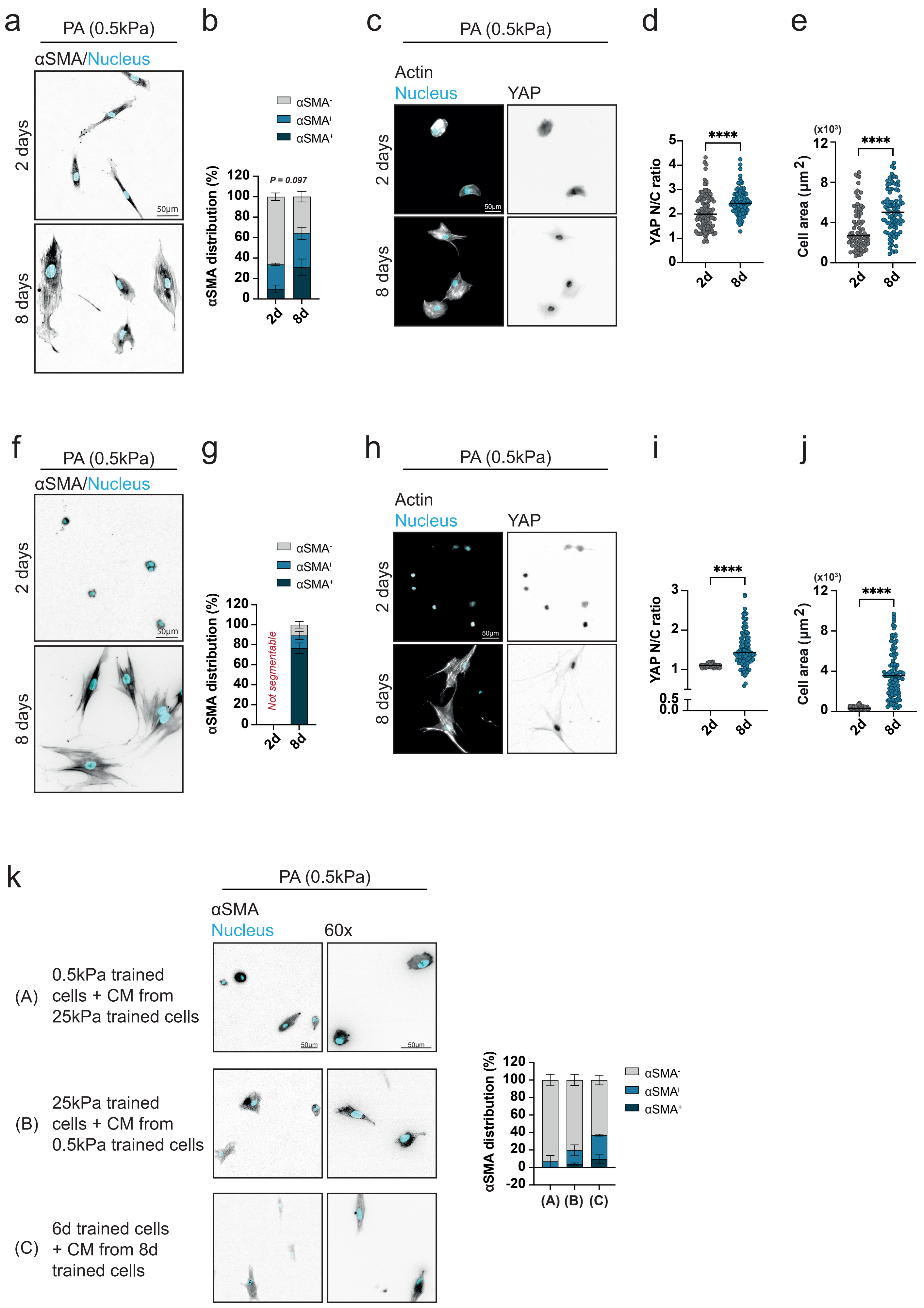
a
PA (0.5kPa)
αSMA/Nucleus
2 days
8 days
50μm
b
αSMA-
αSMAi
αSMA+
αSMA distribution (%)
P = 0.097
2d
8d
c
PA (0.5kPa)
Actin
Nucleus
YAP
2 days
8 days
50μm
d
YAP N/C ratio
****
2d
8d
e
(x10³)
Cell area (μm2)
****
2d
8d
f
PA (0.5kPa)
αSMA/Nucleus
2 days
8 days
50μm
g
αSMA-
αSMAi
αSMA+
αSMA distribution (%)
Not segmentable
2d
8d
h
PA (0.5kPa)
Actin
Nucleus
YAP
2 days
8 days
50μm
i
YAP N/C ratio
****
2d
8d
j
(x10³)
Cell area (μm2)
****
2d
8d
k
PA (0.5kPa)
αSMA
Nucleus
60x
(A) 0.5kPa trained cells + CM from 25kPa trained cells
(B) 25kPa trained cells + CM from 0.5kPa trained cells
(C) 6d trained cells + CM from 8d trained cells
50μm
αSMA distribution (%)
(A) (B) (C)
αSMA-
αSMAi
αSMA+

Figure S2

(a,b) Representative cropped 20x and 60x images of TIFs replated on 0.5kPa hydrogels with 24h conditioned media following culturing on 25kPa hydrogels for different periods of time, showing nucleus (cyan) and αSMA (gray) **(a)** and quantification of αSMA distribution **(b)**. (n = 246 (8d), 156 (2d) cells from 3 experimental repeats). Statistical tests done between αSMA^{+} cells. Welch's t test. Error bars represent mean with SEM. **(c)** Representative cropped 20x images of TIFs replated on 0.5kPa hydrogels with 24h conditioned media following culturing on 25kPa hydrogels for different periods of time, showing actin (gray), nucleus (cyan) and YAP (gray) **(d,e)** Quantification of YAP N/C ratio **(d)** and cell area **(e)** from TIFs replated on 0.5kPa hydrogels with 24h conditioned media following culturing on 25kPa hydrogels for different periods of time. (n = 81-112 (2d), 88-102 (8d) cells from 3 experimental repeats). Mann Whitney test, ****P <0,0001. Bars represent median values. **(f,g)** Representative cropped 20x and 60x images of primary human lung fibroblasts replated on 0.5kPa hydrogels with 24h conditioned media following culturing on 25kPa hydrogels for different periods of time, showing nucleus (cyan) and αSMA (gray) **(f)** and quantification of αSMA distribution **(g)**. (n = 132 (8d) cells from 3 experimental repeats). Error bars represent mean with SEM. **(h)** Representative cropped 20x images of primary human lung fibroblasts replated on 0.5kPa hydrogels with 24h conditioned media following culturing on 25kPa hydrogels for different periods of time, showing actin (gray), nucleus (cyan) and YAP (gray). **(i,j)** Quantification of YAP N/C ratio **(i)** and cell area **(j)** from primary human lung fibroblasts replated on 0.5kPa hydrogels with 24h conditioned media following culturing on 25kPa hydrogels for different periods of time. (n = 86 (2d), 113 (8d) cells from 3 experimental repeats). Mann Whitney test, ****P <0,0001. Bars represent median values. **(k)** Representative cropped 20x and 60x Ti2 images of vCAFs replated on 0.5kPa hydrogels following (A) 8d culture on 0.5Kpa hydrogels with conditioned media from 8d 25Kpa cultured cells, (B) 8d culture on 25kPa hydrogels with conditioned media from 8d 0.5kPa cultured cells and (C) 6d culture on 25kPa hydrogels with conditioned media from 8d 25kPa cultured cells, showing nucleus (cyan) and αSMA (gray). Quantification of αSMA distribution for each condition. (n = 169 (A), 126 (B), 140 (C) cells from 3 experimental repeats). Error bars represent mean with SEM. αSMA^{+}: activated, αSMA^{i}: intermediately activated, αSMA^{-}: not activated cells. PA : persistent activation

Figure S3

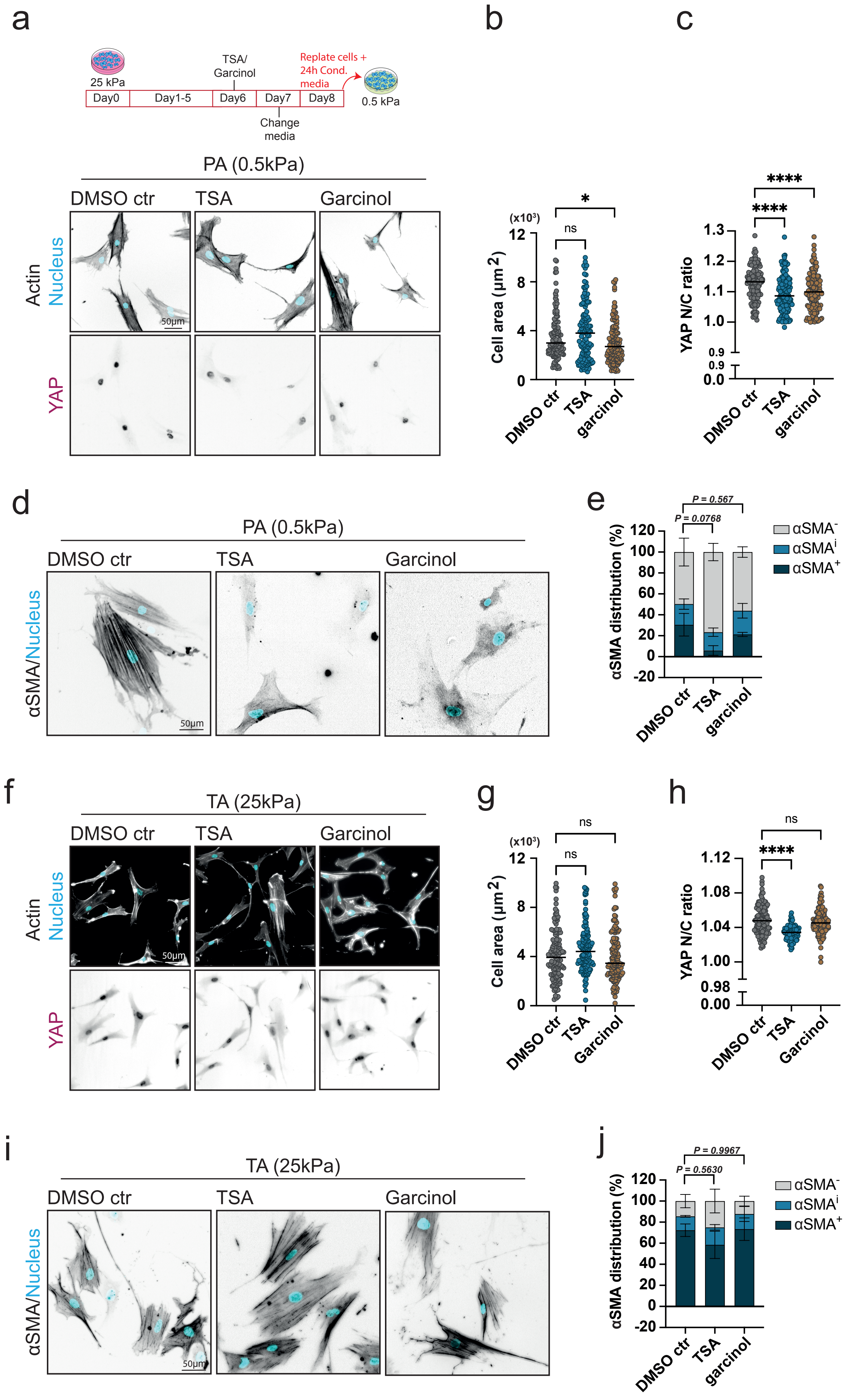
a
25 kPa
Day0
Day1-5
TSA/
Garcinol
Day6
Day7
Day8
Change
media
Replate cells +
24h Cond.
media
0.5 kPa
PA (0.5kPa)
DMSO ctr
TSA
Garcinol
Actin
Nucleus
YAP
50μm
b
(x10³)
Cell area (μm 2)
ns
*
DMSO ctr
TSA
garcinol
c
YAP N/C ratio
****
****
d
αSMA/Nucleus
e
αSMA distribution (%)
P = 0.567
P = 0.0768
αSMA⁻
αSMAⁱ
αSMA⁺
f
TA (25kPa)
g
h
i
j
P = 0.9967
P = 0.5630
Garcinol

Figure S3

(a) Schematic representation of the experimental workflow. Representative cropped 20x images of vCAFs replated on 0.5kPa hydrogels following treatment with TSA, garcinol or DMSO during 8d culture on 25kPa hydrogels showing actin (gray), nucleus (cyan) and YAP (gray). **(b,c)** Quantification of cell area **(b)** and YAP N/C **(c)** from vCAFs replated on 0.5kPa hydrogels following treatment with TSA, garcinol or DMSO during 8d culture on 25kPa hydrogels. (n = 116-134 (DMSO ctr), 117-153 (TSA), 127-142 (garcinol) cells from 3 experimental repeats). Kruskal-Wallis test, *P ≤ 0.05, ****P <0,0001, ns = not significant. Bars represent median values.**(d,e)** Representative cropped 20x images of vCAFs replated on 0.5kPa hydrogels following treatment with TSA, garcinol or DMSO during 8d culture on 25kPa hydrogels showing nucleus (cyan) and αSMA (gray) **(d)** and quantification of αSMA distribution **(e).** (n = 131 (DMSO ctr), 114 (TSA), 118 (garcinol) cells from 3 experimental repeats). Statistical tests done between αSMA^{+} cells. Ordinary one-way ANOVA. Error bars represent mean with SEM. **(f)** Representative cropped 20x images of vCAFs cultured on 25kPa hydrogels for 7d following treatment with TSA, garcinol or DMSO on 6d showing actin (gray), nucleus (cyan) and YAP (gray). **(g,h)** Quantification of cell area **(g)** and YAP N/C **(h)** from vCAFs cultured on 25kPa hydrogels for 7d following treatment with TSA, garcinol or DMSO on 6d. (n = 122 (DMSO ctr), 117 (TSA), 118 (garcinol) cells from 3 experimental repeats). Kruskal-Wallis test, ****P <0,0001, ns = not significant. Bars represent median values. **(i,j)** Representative cropped 20x images of vCAFs cultured on 25kPa hydrogels for 7d following treatment with TSA, garcinol or DMSO on 6d, showing nucleus (cyan) and αSMA (gray) **(i)** and quantification of αSMA distribution **(j).** (n = 114 (DMSO ctr), 132 (TSA), 88 (garcinol)cells from 3 experimental repeats). Statistical tests done between αSMA^{+} cells. Ordinary one-way ANOVA. Error bars represent mean with SEM. αSMA^{+}: activated, αSMA^{i}: intermediately activated, αSMA^{-}: not activated cells. PA : persistent activation, TA : transient activation

Figure S4

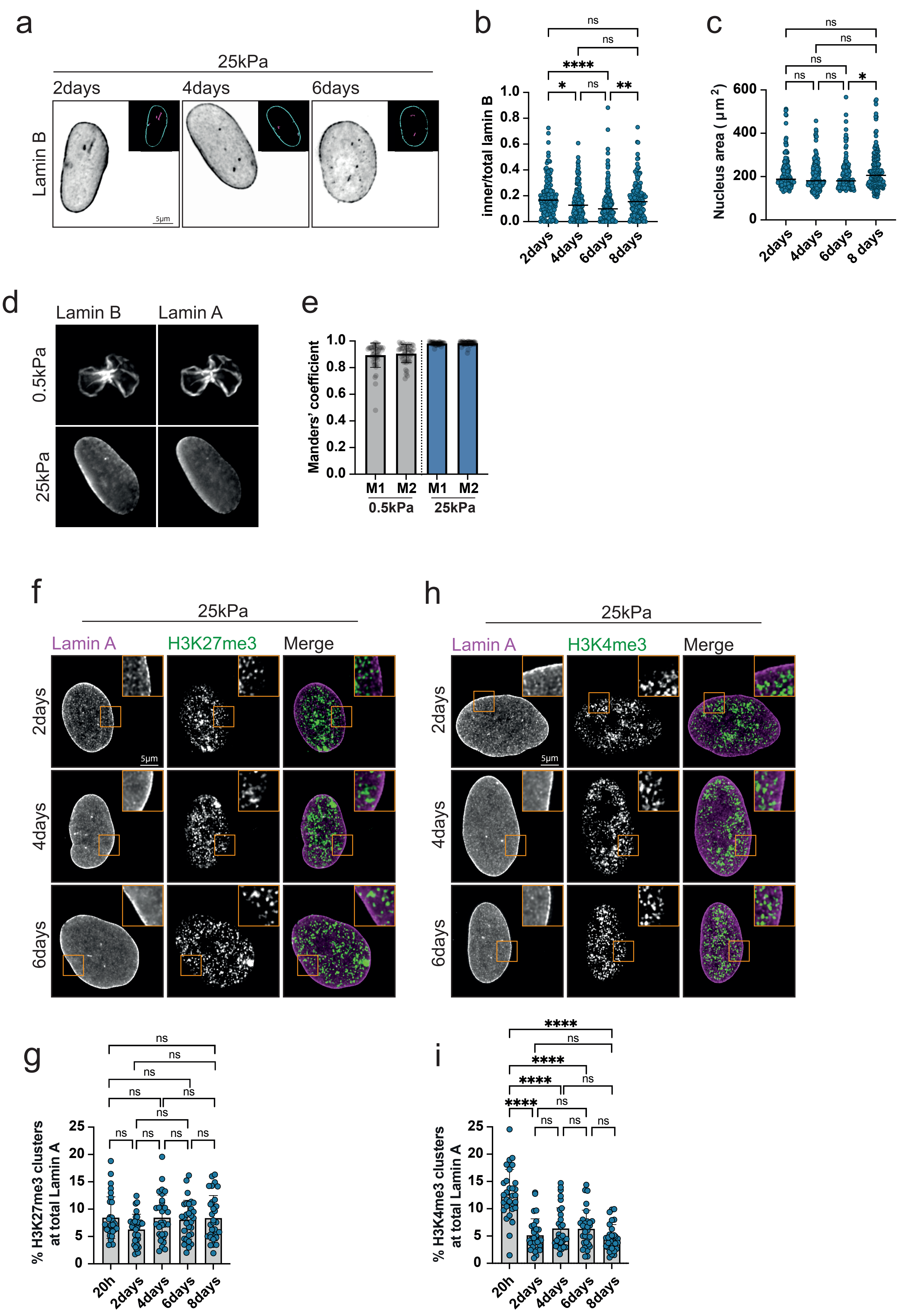

a
25kPa
2days
4days
6days
Lamin B
5μm
b
inner/total lamin B
2days
4days
6days
8days
c
Nucleus area (μm 2)
2days
4days
6days
8 days
d
Lamin B
Lamin A
0.5kPa
25kPa
e
Manders' coefficient
M1
M2
0.5kPa
25kPa
f
25kPa
Lamin A
H3K27me3
Merge
2days
4days
6days
5μm
h
25kPa
Lamin A
H3K4me3
Merge
2days
4days
6days
5μm
g
% H3K27me3 clusters at total Lamin A
20h
2days
4days
6days
8days
i
% H3K4me3 clusters at total Lamin A
20h
2days
4days
6days
8days

Figure S4

(a) Representative cropped deconvolved 60x Ti2 images showing Lamin B (gray) from vCAFs cultured on 25kPa hydrogels for different time points. Inset shows inner (magenta) and outer (cyan) Lamin B segmentations. **(b,c)** Quantification of inner/total lamin B area **(b)** and nuclear area **(c)** from vCAFs cultured on 25kPa hydrogels for different time points. (n = 164 (2days), 153 (4days), 159 (6days), 158 (8days) cells from 3 experimental repeats). Kruskal-Wallis test, *P ≤ 0.05, **P ≤ 0.01, ****P <0,0001, ns = not significant. Bars represent median values. Note: 8d data same as in Fig 2b, 2c. **(d,e)** Representative cropped deconvolved 60x Ti2 images showing Lamin B and Lamin A from vCAFs cultured on 25kPa and 0.5kPa hydrogels for 20h **(d)**. Quantification of Manders' coefficients calculated from Lamin B and Lamin A co-staining **(e)**. (n = 46-48 cells). Error bars represent mean with SD. **(f)** Representative cropped deconvolved 60x confocal images showing Lamin A and H3K27me3 from vCAFs cultured on 25kPa hydrogels for different time points. Merge shows Lamin A (magenta) and H3K27me3 (green). Images are background subtracted. **(g)** Quantification of percentage of H3K27me3 clusters at total Lamin A. Total lamin A is the sum of inner and outer lamin A segmentations. (n = 30-31 cells for each condition from 3 experimental repeats). Kruskal-Wallis test, ns = not significant. Error bars represent mean with SD. Note: 20h and 8d data same as in Fig 2e. **(h)** Representative cropped deconvolved 60x confocal images showing Lamin A and H3K4me3 from vCAFs cultured on 25kPa hydrogels for different time points. Merge shows Lamin A (magenta) and H3K4me3 (green). Images are background subtracted. **(i)** Quantification of percentage of H3K4me3 clusters at total Lamin A. Total lamin A is the sum of inner and outer lamin A segmentations. (n = 30 cells for each condition from 3 experimental repeats). Kruskal-Wallis test, ****P <0,0001, ns = not significant. Error bars represent mean with SD. Note: 20h and 8d data same as in Fig 2g.

Figure S5

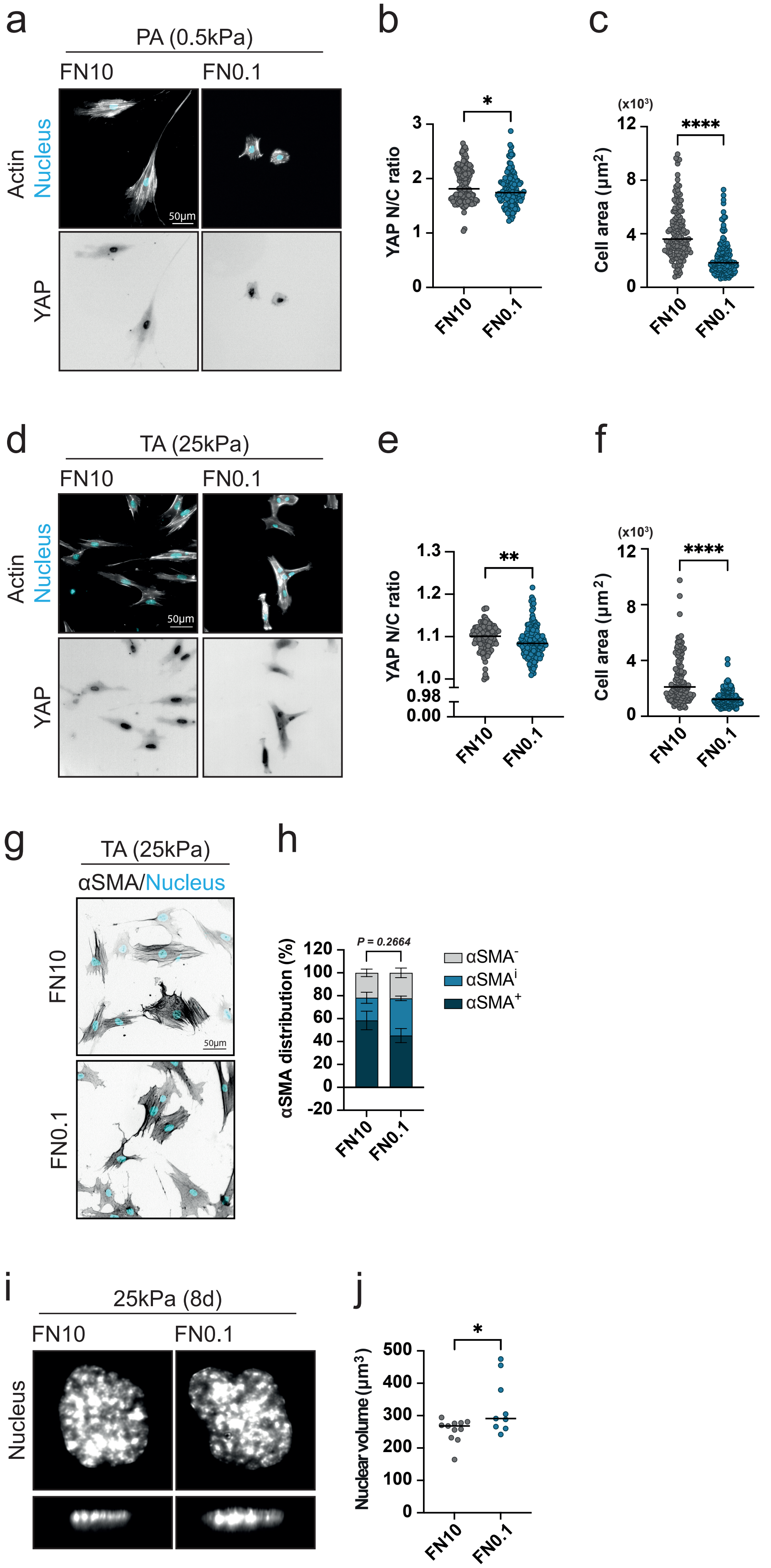
a
PA (0.5kPa)
FN10
FN0.1
Actin
Nucleus
YAP
50μm
b
YAP N/C ratio
*
FN10
FN0.1
c
(x10³)
Cell area (μm²)
****
d
TA (25kPa)
e
**
f
g
αSMA/Nucleus
h
αSMA distribution (%)
P = 0.2664
αSMA⁻
αSMAⁱ
αSMA⁺
i
25kPa (8d)
Nucleus
j
Nuclear volume (μm³)

Figure S5

(a) Representative cropped 20x images of vCAFs replated on 0.5kPa hydrogels following 8d culture on 25kPa hydrogels coated with either 10mg/ml or 0.1mg/ml of FN, showing actin (gray), nucleus (cyan) and YAP (gray). **(b,c)** Quantification of YAP N/C **(b)** and cell area **(c)** from vCAFs replated on 0.5kPa hydrogels following 8d culture on 25kPa hydrogels coated with either 10mg/ml or 0.1mg/ml of FN. (n = 139-143 (FN10), 132-134 (FN0.1) cells from 3 experimental repeats). Mann Whitney test, *P ≤ 0.05, ****P <0,0001. Bars represent median values. **(d)** Representative cropped 20x images of vCAFs cultured on 25kPa hydrogels coated with either 10mg/ml or 0.1mg/ml of FN for 20h, showing actin (gray), nucleus (cyan) and YAP (gray). **(e,f)** Quantification of YAP N/C **(e)** and cell area **(f)** from vCAFs cultured on 25kPa hydrogels coated with either 10mg/ml or 0.1mg/ml of FN for 20h. (n = 120 (FN10), 132 (FN0.1) cells from 3 experimental repeats). Mann Whitney test, **P ≤ 0.01, ****P <0,0001. Bars represent median values. **(g,h)** Representative cropped 20x images of vCAFs cultured on 25kPa hydrogels coated with either 10mg/ml or 0.1mg/ml of FN for 20h, showing nucleus (cyan) and αSMA (gray) **(g)** and quantification of αSMA distribution **(h).** (n = 210 (FN10), 277 (FN0.1) cells from 3 experimental repeats). Statistical tests done between αSMA^{+} cells. Welch's t test. Error bars represent mean with SEM. **(i,j)** Representative cropped 60x confocal images of the nucleus from cells plated on 25kPa hydrogels coated with either 10mg/ml or 0.1mg/ml of FN for 8d with their xz projections shown below **(i)** and quantification of nuclear volume **(j)**. (n = 11 (FN10), 9 (FN01) cells). Mann Whitney test, *P ≤ 0.05. Bars represent median values. αSMA^{+}: activated, αSMA^{i}: intermediately activated, αSMA^{-}: not activated cells. TA : transient activation

Figure S6

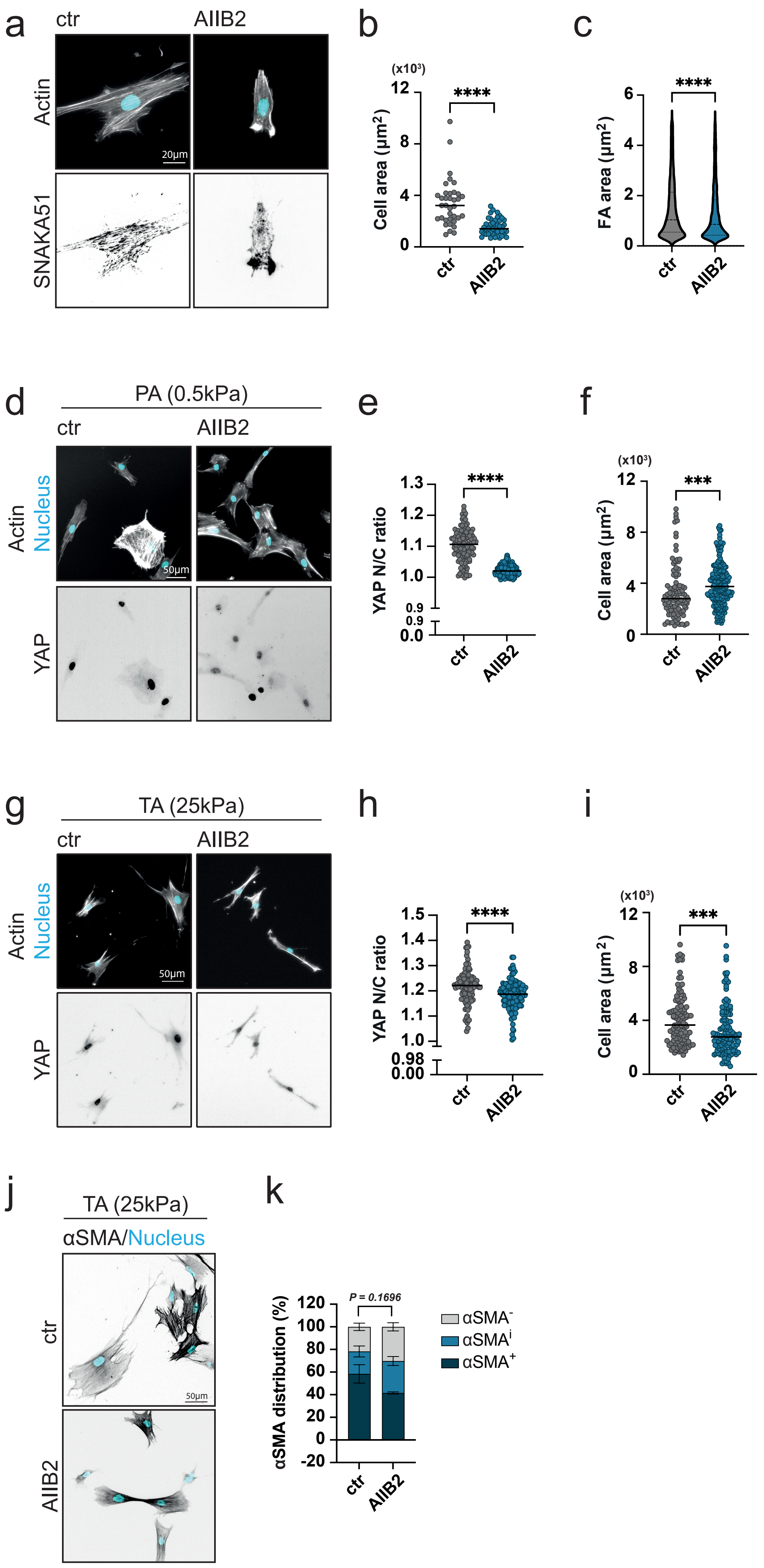

a
ctr
AIIB2
Actin
SNAKA51
20µm
b
(x10³)
Cell area (µm²)
****
ctr
AIIB2
c
FA area (µm²)
****
ctr
AIIB2
d
PA (0.5kPa)
ctr
AIIB2
Actin Nucleus
YAP
50µm
e
YAP N/C ratio
****
ctr
AIIB2
f
(x10³)
Cell area (µm²)
***
ctr
AIIB2
g
TA (25kPa)
ctr
AIIB2
Actin Nucleus
YAP
50µm
h
YAP N/C ratio
****
ctr
AIIB2
i
(x10³)
Cell area (µm²)
***
ctr
AIIB2
j
TA (25kPa)
αSMA/Nucleus
ctr
AIIB2
50µm
k
P = 0.1696
αSMA distribution (%)
αSMA⁻
αSMAⁱ
αSMA⁺
ctr
AIIB2

Figure S6

(a) Representative cropped 20x images of vCAFs treated with AIIB2 and control cells plated on glass for 20h, showing actin (gray), nucleus (cyan) and α5 integrins (gray). **(b,c)** Quantification of cell area **(b)** and FA area **(c)** from vCAFs treated with AIIB2 and control cells plated on glass for 20h. (n = 37 (ctr), 44 (AIIB2) cells) (n = 3569 (ctr), 2858 (AIIB2) FAs). Mann Whitney test, ****P <0,0001. Bars represent median values. **(d)** Representative cropped 20x images of vCAFs replated on 0.5kPa hydrogels following treatment with AIIB2 during 8d culture on 25kPa hydrogels and control, showing actin (gray), nucleus (cyan) and YAP (gray). **(e,f)** Quantification of YAP N/C **(e)** and cell area **(f)** from vCAFs replated on 0.5kPa hydrogels following treatment with AIIB2 during 8d culture on 25kPa hydrogels and control. (n = 98-126 (ctr), 186-233 (AIIB2) cells from 3 experimental repeats). Mann Whitney test, ***P ≤ 0.001, ****P <0,0001. Bars represent median values. Note: AIIB2 data same as in Fig 5f, 5g. **(g)** Representative cropped 20x images of vCAFs cultured on 25kPa hydrogels for 20h treated with AIIB2 and control, showing actin (gray), nucleus (cyan) and YAP (gray). **(h,i)** Quantification of YAP N/C **(h)** and cell area **(i)** from vCAFs cultured on 25kPa hydrogels for 20h treated with AIIB2 and control. (n = 109 (ctr), 115 (AIIB2) cells from 3 experimental repeats). Mann Whitney test, ***P ≤ 0.001, ****P <0,0001. Bars represent median values. **(j,k)** Representative cropped 20x images of vCAFs cultured on 25kPa hydrogels for 20h treated with AIIB2 and control, showing nucleus (cyan) and αSMA (gray) **(j)** and quantification of αSMA distribution **(k).** (n = 210 (ctr), 213 (AIIB2) cells from 3 experimental repeats). Statistical tests done between αSMA^+ cells. Welch's t test. Error bars represent mean with SEM. Note: ctr is same as FN10 in Fig S5h. αSMA^+: activated, αSMA^i: intermediately activated, αSMA^-: not activated cells. PA : persistent activation, TA : transient activation

Figure S7

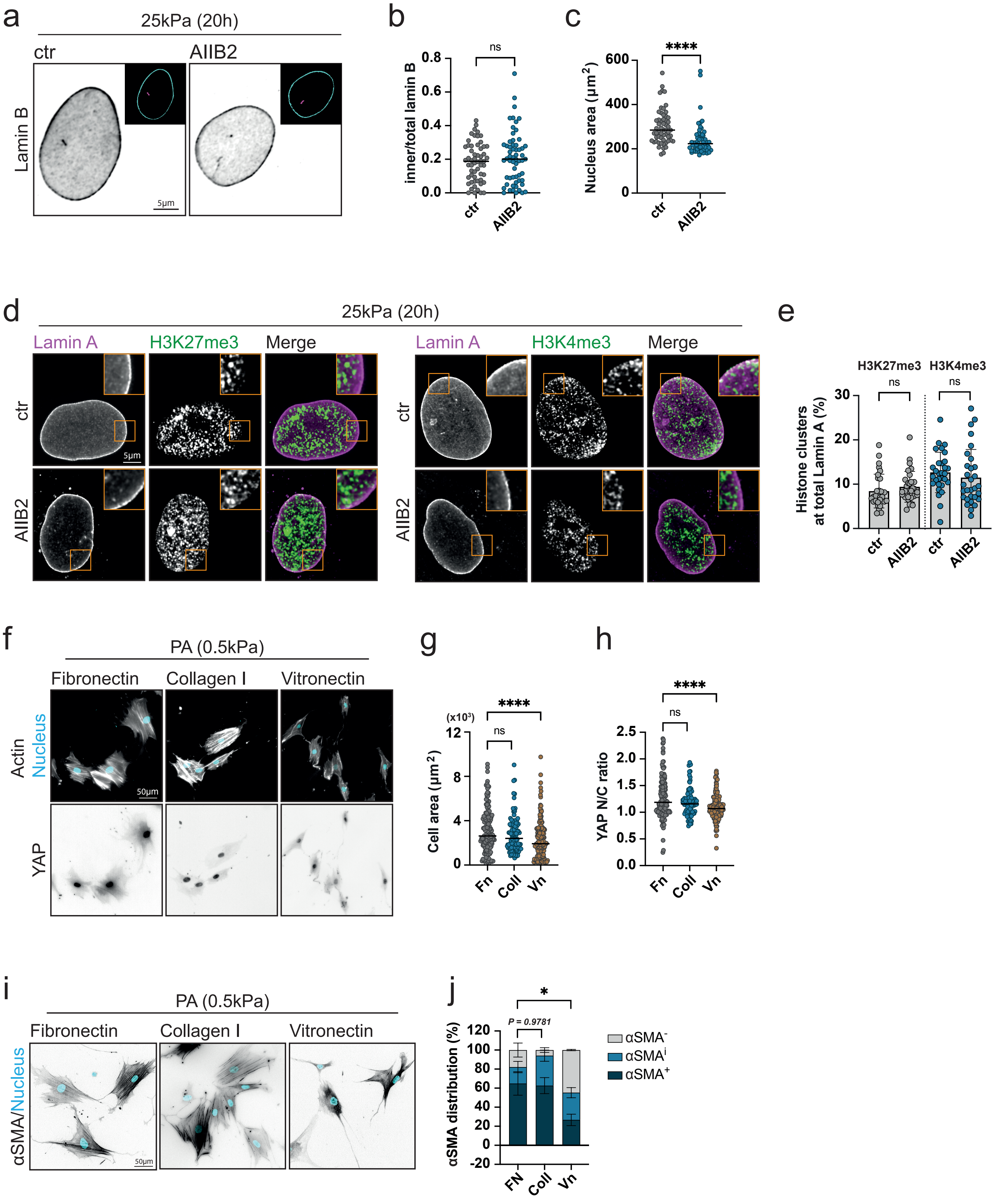

a
25kPa (20h)
ctr
AIIB2
Lamin B
5µm
b
inner/total lamin B
ns
0.8
0.6
0.4
0.2
0.0
ctr
AIIB2
c
Nucleus area (µm²)
****
600
400
200
0
ctr
AIIB2
d
25kPa (20h)
Lamin A
H3K27me3
Merge
Lamin A
H3K4me3
Merge
ctr
AIIB2
5µm
e
H3K27me3
H3K4me3
ns
ns
Histone clusters at total Lamin A (%)
30
20
10
0
ctr
AIIB2
ctr
AIIB2
f
PA (0.5kPa)
Fibronectin
Collagen I
Vitronectin
Actin
Nucleus
YAP
50µm
g
(x10³)
Cell area (µm²)
****
ns
12
8
4
0
Fn
Coll
Vn
h
YAP N/C ratio
****
ns
2.5
2.0
1.5
1.0
0.5
0.0
Fn
Coll
Vn
i
PA (0.5kPa)
Fibronectin
Collagen I
Vitronectin
αSMA/Nucleus
50µm
j
αSMA distribution (%)
*
P = 0.9781
120
100
80
60
40
20
0
-20
FN
Coll
Vn
αSMA⁻
αSMAⁱ
αSMA⁺

Figure S7

(a) Representative cropped deconvolved 60x Ti2 images showing Lamin B (gray) from vCAFs cultured on 25kPa hydrogels for 20h treated with AIIB2 and control. Inset shows inner (magenta) and outer (cyan) Lamin B segmentations. **(b,c)** Quantification of inner/total lamin B area **(b)** and nuclear area **(c)** from vCAFs cultured on 25kPa hydrogels for 20h treated with AIIB2 and control. (n = 57 (ctr), 59 (AIIB2) cells from 3 experimental repeats). Mann Whitney test, ****P <0,0001, ns = not significant. Bars represent median values. Note: 20h ctr data same as 20h 25kPa data in Fig 2b, 2c. **(d)** Representative cropped deconvolved 60x confocal images showing Lamin A and H3K27me3 (left) and H3K4me3 (right) from vCAFs cultured on 25kPa for 20h treated with AIIB2 and control. Merge shows Lamin A (magenta) and H3K27me3/H3K4me3 (green). Images are background subtracted. **(e)** Quantification of percentage of H3K27me3 and H3K4me3 clusters at total Lamin A. Total lamin A is the sum of inner and outer lamin A segmentations. (n = 30 cells for each condition from 3 experimental repeats). Mann Whitney test, ns = not significant. Error bars represent mean with SD. Note: 20h ctr data same as 20h data in Fig 2e and 2g. **(f)** Representative cropped 20x images of vCAFs replated on 0.5kPa hydrogels following 8d culture on 25kPa hydrogels coated with fibronectin, collagen I or vitronectin, showing actin (gray), nucleus (cyan) and YAP (gray). **(g,h)** Quantification of cell area **(g)** and YAP N/C **(h)** from vCAFs vCAFs replated on 0.5kPa hydrogels following 8d culture on 25kPa hydrogels coated with fibronectin, collagen I or vitronectin. (n = 141 (Fibronectin), 102 (collagen I), 163 (Vitronectin) cells from 3 experimental repeats). Kruskal-Wallis test, ****P <0,0001, ns = not significant. Bars represent median values. **(i,j)** Representative cropped 20x images of vCAFs replated on 0.5kPa hydrogels following 8d culture on 25kPa hydrogels coated with fibronectin, collagen I or vitronectin, showing nucleus (cyan) and αSMA (gray) **(i)** and quantification of αSMA distribution **(j).** (n = 96 (FN), 137 (VN), 243 (collagen) cells from 3 experimental repeats). Statistical tests done between αSMA^+ cells. Ordinary one-way ANOVA, *P ≤ 0.05. Error bars represent mean with SEM. αSMA^+: activated, αSMA^i: intermediately activated, αSMA^-: not activated cells. PA : persistent activation

Figure S8

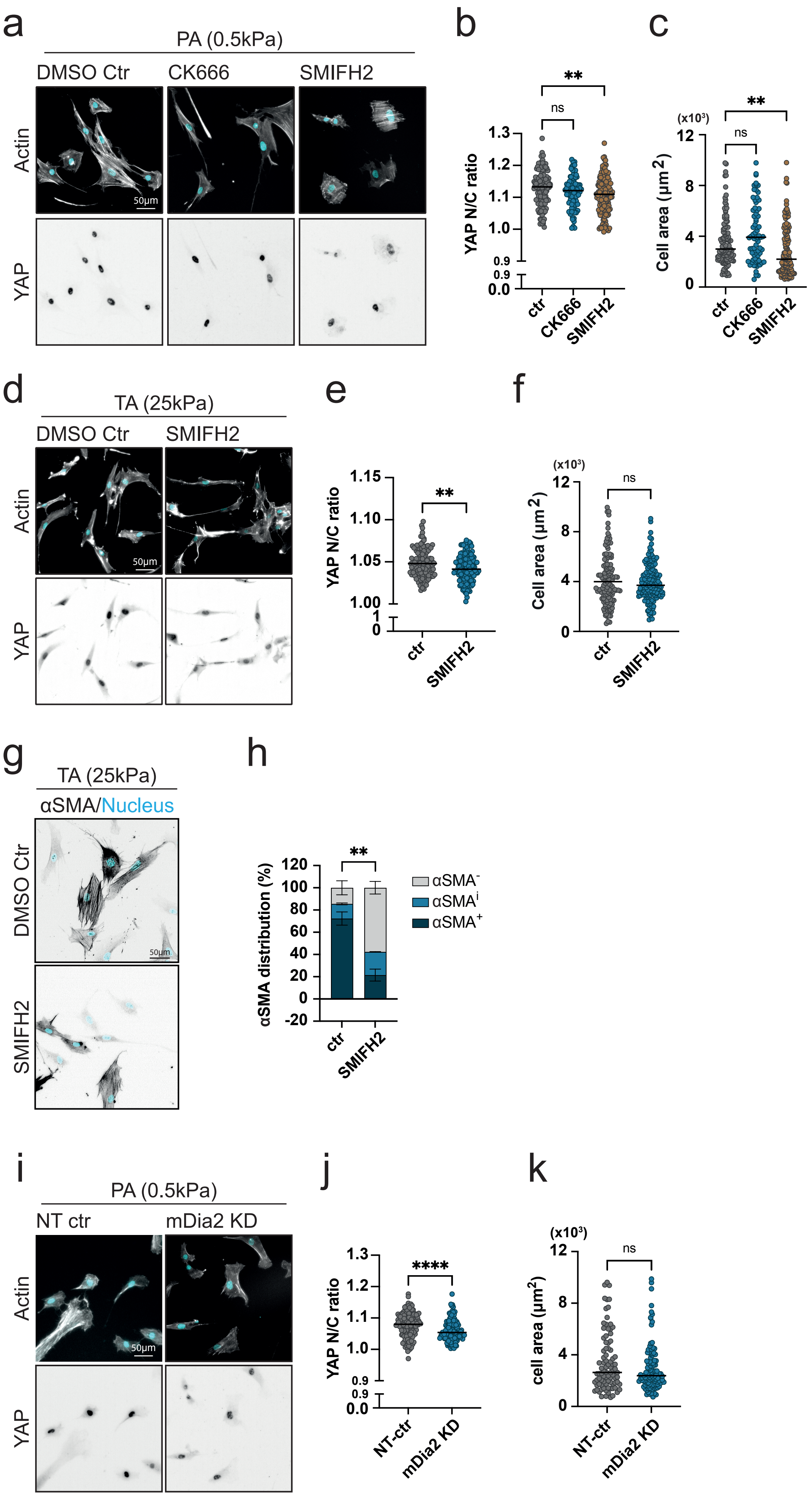

a
PA (0.5kPa)
DMSO Ctr
CK666
SMIFH2
Actin
YAP
50µm
b
YAP N/C ratio
ns
**
ctr
CK666
SMIFH2
c
(x10³)
Cell area (µm²)
ns
**
ctr
CK666
SMIFH2
d
TA (25kPa)
DMSO Ctr
SMIFH2
Actin
YAP
50µm
e
YAP N/C ratio
**
ctr
SMIFH2
f
(x10³)
Cell area (µm²)
ns
ctr
SMIFH2
g
TA (25kPa)
αSMA/Nucleus
DMSO Ctr
SMIFH2
50µm
h
αSMA distribution (%)
**
αSMA⁻
αSMAⁱ
αSMA⁺
ctr
SMIFH2
i
PA (0.5kPa)
NT ctr
mDia2 KD
Actin
YAP
50µm
j
YAP N/C ratio
****
NT-ctr
mDia2 KD
k
(x10³)
cell area (µm²)
ns
NT-ctr
mDia2 KD

Figure S8

(a) Representative cropped 20x images of vCAFs replated on 0.5kPa hydrogels following treatment with CK666, SMIFh2 or DMSO during 8d culture on 25kPa hydrogels, showing actin (gray), nucleus (cyan) and YAP (gray). **(b,c)** Quantification of YAP N/C **(b)** and cell area **(c)** from vCAFs replated on 0.5kPa hydrogels following treatment with CK666, SMIFh2 or DMSO during 8d culture on 25kPa hydrogels. (n = 116-134 (DMSO ctr), 73-82 (CK666), 119-135 (SMIFh2) cells from 3 experimental repeats). Kruskal Wallis test, $^{**}P \leq 0.01$, ns = not significant. Bars represent median values. Note: ctr data same as in Fig S3b, S3c. **(d)** Representative cropped 20x images of vCAFs cultured on 25kPa hydrogels and treated with SMIFh2 or DMSO, showing actin (gray), nucleus (cyan) and YAP (gray). **(e,f)** Quantification of YAP N/C **(e)** and cell area **(f)** from vCAFs cultured on 25kPa hydrogels and treated with SMIFh2 or DMSO. (n = 119 (DMSO ctr), 144 (SMIFh2) cells from 3 experimental repeats). Mann Whitney test, $^{**}P \leq 0.01$, ns = not significant. Bars represent median values. **(g,h)** Representative cropped 20x images of vCAFs cultured on 25kPa hydrogels and treated with SMIFh2 or DMSO, showing nucleus (cyan) and αSMA (gray) **(g)** and quantification of αSMA distribution **(h)**. (n = 115 (DMSO ctr), 226 (SMIFH2) cells from 3 experimental repeats). Statistical tests done between αSMA^{+} cells. Welch's t test, $^{**}P \leq 0.01$. Error bars represent mean with SEM. Note: DMSO ctr same as in S3j. **(i)** Representative cropped 20x images of NT-ctr and mDia2 KD vCAFs replated on 0.5kPa hydrogels following 8d culture on 25kPa hydrogels, showing actin (gray), nucleus (cyan) and YAP (gray). **(j,k)** Quantification of YAP N/C **(j)** and cell area **(k)** from NT-ctr and mDia2 KD vCAFs replated on 0.5kPa hydrogels following 8d culture on 25kPa hydrogels. (n = 88-129 (NT-ctr), 105-137 (mDia2 KD) cells from 3 experimental repeats). Mann Whitney test, $^{****}P < 0{,}0001$, ns = not significant. Bars represent median values. Note: mDia2 KD data same as in Fig 5i, 5j. αSMA^{+} : activated, αSMA^{i} : intermediately activated, αSMA^{-} : not activated cells. PA : persistent activation, TA : transient activation

Figure S9

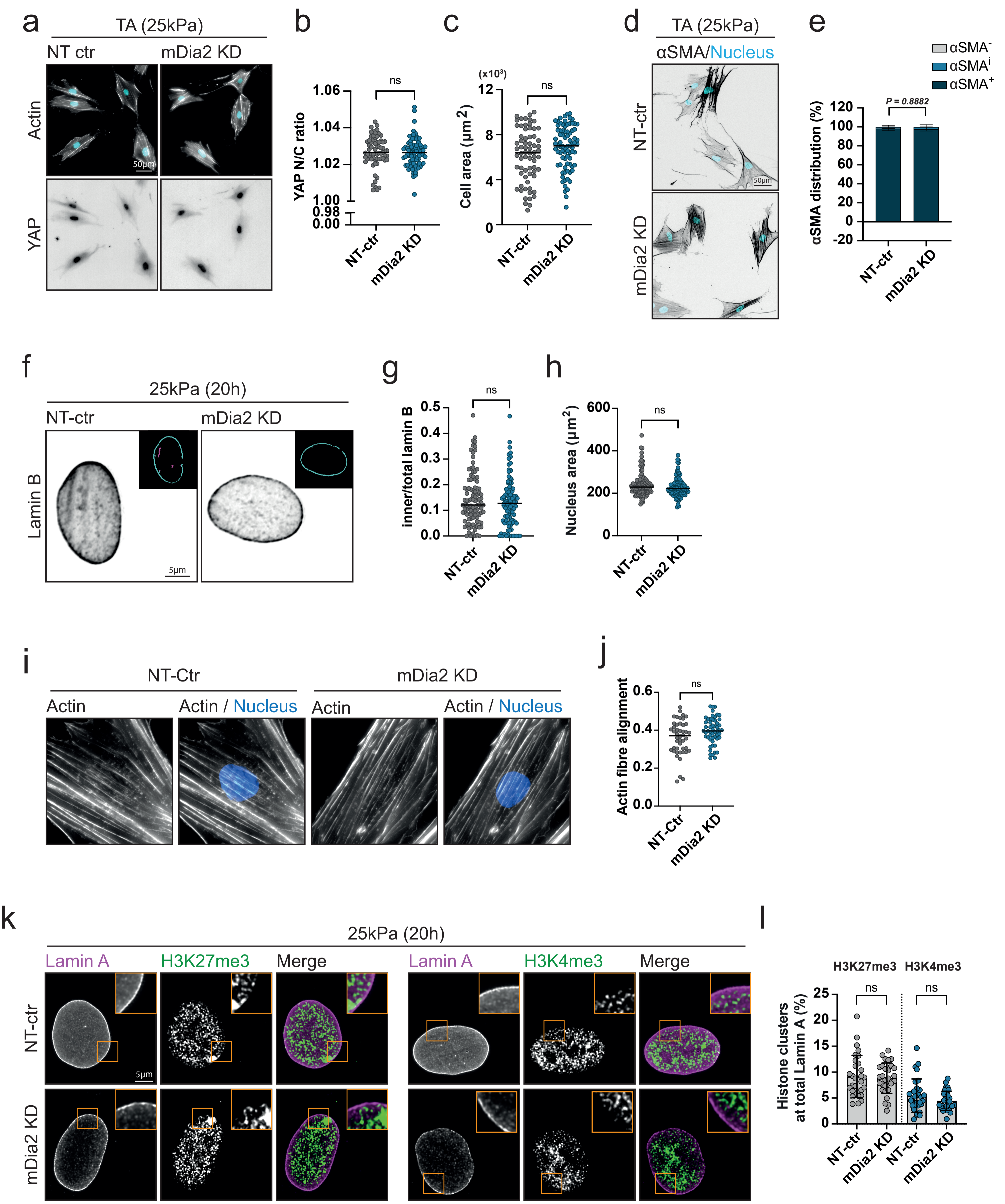

a
TA (25kPa)
NT ctr
mDia2 KD
Actin
YAP
50µm
b
YAP N/C ratio
ns
NT-ctr
mDia2 KD
c
Cell area (µm²)
(x10³)
ns
NT-ctr
mDia2 KD
d
TA (25kPa)
αSMA/Nucleus
NT-ctr
mDia2 KD
50µm
e
αSMA⁻
αSMAⁱ
αSMA⁺
P = 0.8882
αSMA distribution (%)
NT-ctr
mDia2 KD
f
25kPa (20h)
NT-ctr
mDia2 KD
Lamin B
5µm
g
inner/total lamin B
ns
NT-ctr
mDia2 KD
h
Nucleus area (µm²)
ns
NT-ctr
mDia2 KD
i
NT-Ctr
mDia2 KD
Actin
Actin / Nucleus
j
Actin fibre alignment
ns
NT-Ctr
mDia2 KD
k
25kPa (20h)
Lamin A
H3K27me3
Merge
H3K4me3
NT-ctr
mDia2 KD
5µm
l
H3K27me3
H3K4me3
Histone clusters at total Lamin A (%)
ns
NT-ctr
mDia2 KD

Figure S9

(a) Representative cropped 20x images of NT-ctr and mDia2 KD vCAFs cultured on 25kPa hydrogels for 20h, showing actin (gray), nucleus (cyan) and YAP (gray). **(b,c)** Quantification of YAP N/C **(b)** and cell area **(c)** from NT-ctr and mDia2 KD vCAFs cultured on 25kPa hydrogels for 20h. (n = 70 (NT-ctr r), 81 (mDia2 KD) cells from 3 experimental repeats). Mann Whitney test, ns = not significant. Bars represent median values. **(d,e)** Representative cropped 20x images of NT-ctr and mDia2 KD vCAFs cultured on 25kPa hydrogels for 20h, showing nucleus (cyan) and αSMA (gray) **(d)** and quantification of αSMA distribution **(e).** (n = 88 (NT-ctr), 109 (mDia2 KD) cells from 3 experimental repeats). Statistical tests done between αSMA^{+} cells. Welch's t test. Error bars represent mean with SEM. **(f)** Representative cropped deconvolved 60x Ti2 images showing Lamin B (gray) from NT-ctr and mDia2 KD cells cultured on 25kPa hydrogels for 20h. Inset shows inner (magenta) and outer (cyan) Lamin B segmentations. **(g,h)** Quantification of inner/total lamin B area **(g)** and nuclear area **(h)** from NT-ctr and mDia2 KD cells cultured on 25kPa hydrogels for 20h. (n = 102 (NT-ctr), 106 (mDia2 KD) cells from 2 experimental repeats). Mann Whitney test, ns = not significant. Bars represent median values. **(i,j)** Representative cropped 60x Ti2 images of NT-Ctr and mDia2 KD vCAFs plated on 25kPa hydrogels for 20h showing actin (gray) and nucleus (blue) **(i)** and quantification of actin fiber alignment around the nucleus **(j)**. (n = 47 (NT-Ctr), 54 (mDia2 KD) cells from 2 experimental repeats). Mann Whitney test, ns = not significant. Bars represent median values. **(k)** Representative cropped deconvolved 60x confocal images showing Lamin A and H3K27me3 (left) or H3K4me3 (right) from NT-ctr and mDia2 KD cells cultured on 25kPa for 20h. Merge shows Lamin A (magenta) and H3K27me3/H3K4me3 (green). Images are background subtracted. **(l)** Quantification of percentage of H3K27me3 and H3K4me3 clusters at total Lamin A. Total lamin A is the sum of inner and outer lamin A segmentations. (n = 29-30 cells for each condition from 3 experimental repeats). Mann Whitney test, ns = not significant. Error bars represent mean with SD. αSMA^{+}: activated, αSMA^{i}: intermediately activated, αSMA^{-}: not activated cells. TA : transient activation

Figure S10

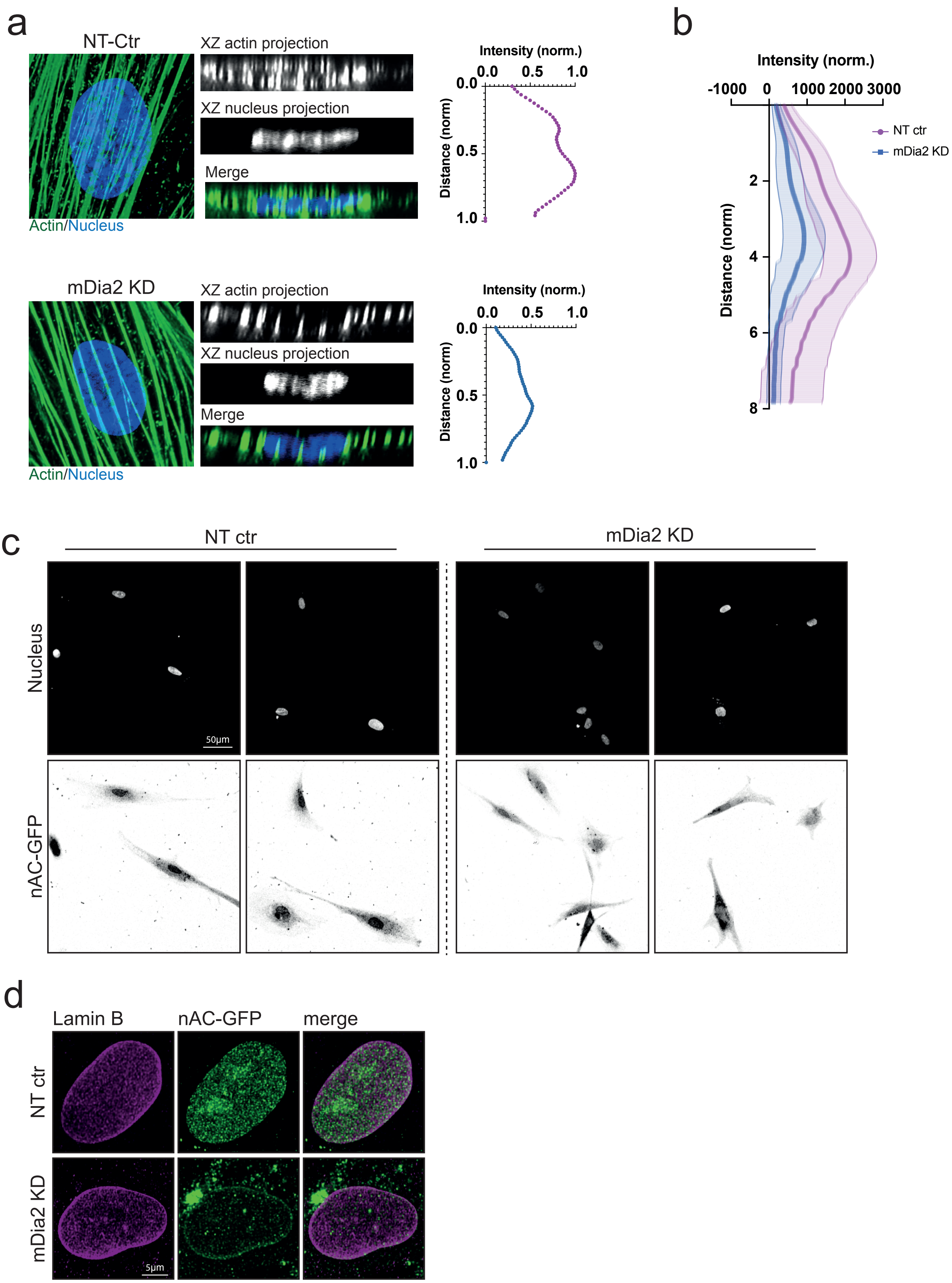
a
NT-Ctr
XZ actin projection
XZ nucleus projection
Merge
Actin/Nucleus
Intensity (norm.)
0.0 0.5 1.0
Distance (norm)
0.0
0.5
1.0
mDia2 KD
XZ actin projection
XZ nucleus projection
Merge
Actin/Nucleus
Intensity (norm.)
0.0 0.5 1.0
Distance (norm)
0.0
0.5
1.0
b
Intensity (norm.)
-1000 0 1000 2000 3000
NT ctr
mDia2 KD
Distance (norm)
2
4
6
8
c
NT ctr
mDia2 KD
Nucleus
nAC-GFP
50µm
d
Lamin B
nAC-GFP
merge
NT ctr
mDia2 KD
5µm

Figure S10

(a) 60x confocal images of NT-ctr and mDia2 KD vCAFs cultured on 25kPa for 20h, showing actin (green) and nucleus (blue) with xz projections and representative line scans drawn across xz projections of actin in regions of the nucleus. **(b)** Line scan plot showing the average of multiple line scans drawn across xy projections of actin in regions of the nucleus from multiple cells. (n = 20 cells from 2 experimental repeats). **(c,d)** Representative cropped 20x confocal images of NT-ctr and mDia2 KD vCAFs transfected with nuclear actin chromobody cultured on glass for 20h, showing nucleus (above) and nAC-GFP (below) **(c)** and 60x confocal images of Lamin B (magenta) and nAC-GFP (green) **(d)**.

Figure S11

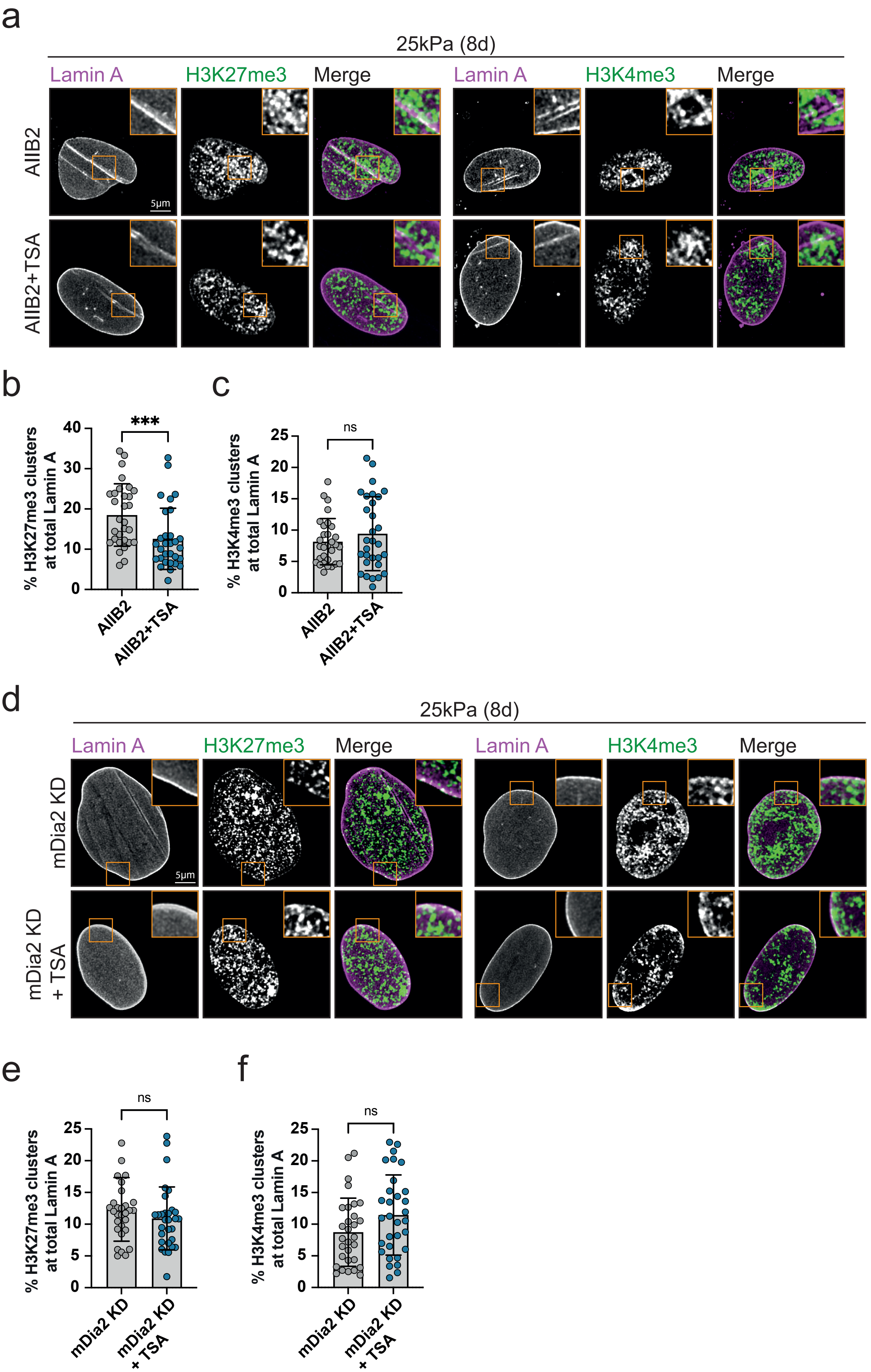
a
25kPa (8d)
Lamin A
H3K27me3
Merge
Lamin A
H3K4me3
Merge
AIIB2
AIIB2+TSA
5µm
b
***
% H3K27me3 clusters at total Lamin A
AIIB2
AIIB2+TSA
c
ns
% H3K4me3 clusters at total Lamin A
AIIB2
AIIB2+TSA
d
25kPa (8d)
Lamin A
H3K27me3
Merge
Lamin A
H3K4me3
Merge
mDia2 KD
mDia2 KD + TSA
5µm
e
ns
% H3K27me3 clusters at total Lamin A
mDia2 KD
mDia2 KD + TSA
f
ns
% H3K4me3 clusters at total Lamin A
mDia2 KD
mDia2 KD + TSA

Figure S11

(a) Representative cropped deconvolved 60x confocal images showing Lamin A and H3K27me3 (left) and H3K4me3 (right) from AIIB2 and AIIB2 + TSA treated vCAFs cultured on 25kPa for 8d. Merge shows Lamin A (magenta) and H3K27me3/H3K4me3 (green). Images are background subtracted. **(b,c)** Quantification of percentage of H3K27me3 clusters at total Lamin A **(b)** and percentage of H3K4me3 clusters at total Lamin A **(c)** Total lamin A is the sum of inner and outer lamin A segmentations. (n = 30 cells for each condition from 3 experimental repeats). Mann Whitney test, ***P ≤ 0.001, ns = not significant. Error bars represent mean with SD. **(d)** Representative cropped deconvolved 60x confocal images showing Lamin A and H3K27me3 (left) and H3K4me3 (right) from mDia2 KD and mDia2 KD + TSA vCAFs cultured on 25kPa for 8d. Merge shows Lamin A (magenta) and H3K27me3/H3K4me3 (green). Images are background subtracted. **(e,f)** Quantification of percentage of H3K27me3 clusters at total Lamin A **(e)** and percentage of H3K4me3 clusters at total Lamin A **(f)** Total lamin A is the sum of inner and outer lamin A segmentations. (n = 30-31 cells for each condition from 3 experimental repeats). Mann Whitney test, ns = not significant. Error bars represent mean with SD.

Figure S12

a

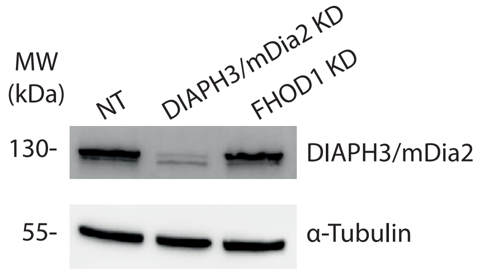

b

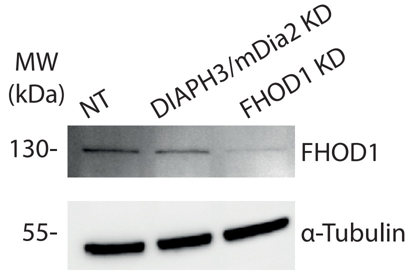

Figure S12

(a) Representative Western blotting images of cell lysates from vCAFs showing DIAPH3/mDia2 and α-Tubulin 48 h post transfection with non-targeting (NT), DIAPH3/mDia2 or FHOD1 siRNA. **(b)** Representative Western blotting images of cell lysates from vCAFs showing FHOD1 and α-Tubulin 48 h post transfection with non-targeting (NT), DIAPH3/mDia2 or FHOD1 siRNA.
